# Palaeomagnetic and geochronologic results from lower cretaceous volcanics of the western Qiangtang terrane and implications for the Lhasa-Qiangtang collision

**DOI:** 10.1038/s41598-025-30138-7

**Published:** 2025-11-27

**Authors:** Yabo Zhang, Weiwei Bian, Jiahui Ma, Suo Wang, Xianwei Jiao, Jiacheng Liang, Siqi Wang, Xiaolin Li, Jikai Ding, Hanqing Zhao, Haiyan Li, Huaichun Wu, Yiming Ma, Tianshui Yang

**Affiliations:** 1 State Key Laboratory of Geomicrobiology and Environmental Changes, China University of Geosciences, Beijing, China; 2 State Key Laboratory of Lithospheric and Environmental Coevolution, Institute of Geology and Geophysics, Chinese Academy of Sciences, Beijing, China; 3 Key Lab of Submarine Geosciences and Prospecting Techniques, Ocean University of China, Qingdao, China; 4 Beijing Institute of Geological Survey, Beijing, China; 5 School of Earth Sciences, China University of Geosciences, Wuhan, Hubei China

**Keywords:** Climate sciences, Solid Earth sciences

## Abstract

The Lhasa-Qiangtang collision is key to understanding the formation and evolution of the Tibetan Plateau; however, the precise timing of this event remains hotly debated. To address this uncertainty, we present a combined palaeomagnetic and geochronologic study of Lower Cretaceous Meiriqieco Formation rhyolites from the western Qiangtang terrane. Our geochronologic data reveal an Early Cretaceous (ca. 113–109) age for the Meiriqieco Formation, contradicting previous Late Jurassic assignments. The tilt-corrected site-mean direction yields a palaeopole at 74.3°N, 250.5°E with A_95_ = 4.7° (*N* = 23). Integrating our new and existing palaeomagnetic data, we place the western Qiangtang terrane at ~ 23.1°N during the Early Cretaceous. Comparison with Early Cretaceous palaeomagnetic data from the western Lhasa terrane indicates that convergence of the two terranes occurred no later than the Early Cretaceous (ca. 132–100 Ma).

**Supplementary Information:**

The online version contains supplementary material available at 10.1038/s41598-025-30138-7.

## Introduction

The Tibetan Plateau, known as the Earth’s “third pole”, comprises a series of continental blocks (Qiangtang, Lhasa, and Himalaya) that rifted from Gondwana and subsequently collided with Eurasia since the Palaeozoic. The tectonic evolution of the plateau was accompanied by the opening and closing of the Palaeo-, Meso-, and Neo-Tethys oceans^1^. Given the tremendous impact of these processes on palaeogeography, palaeoenvironment, and palaeoclimate, understanding the formation and evolution of the Tibetan Plateau is crucial for advancing our knowledge of plate tectonic kinematics and climate change^2–4^.

The India-Asia and Lhasa-Qiangtang collisions ultimately led to the closure of the Neo- and Meso-Tethys oceans, respectively^5,6^. Although the Cenozoic (starting around ~ 60–55 Ma) India-Asia collision and subsequent continuous convergence fundamentally controlled the phased uplift of the Tibetan Plateau^3^, recent research emphasizes the significant contribution of the late Mesozoic Lhasa-Qiangtang collision to the plateau’s early development^7–10^. However, estimates of the timing of the initial Lhasa-Qiangtang collision remain highly variable^6,11^. Some researchers propose a Middle Jurassic age^12,13^, while others favor a Late Jurassic-Early Cretaceous interval^14–17^, and still others suggest that the collision did not occur until the Late Cretaceous^18,19^. In addition, some researchers propose a diachronous collision, with initiation in the east around ∼162 Ma and westward propagation continuing until the mid-Cretaceous^20^. In contrast, others argue for a synchronous collision during the Cretaceous^21,22^.

Palaeomagnetism, the only method for quantifying palaeolatitude, is crucial for understanding terrane movement. Palaeogeographic reconstructions of the Lhasa terrane are well-defined by abundant Cretaceous and Palaeocene palaeomagnetic data^23–37^. However, few Early Cretaceous palaeomagnetic data exist for the Qiangtang terrane^38–43^. Two Early Cretaceous palaeopoles from red beds in the eastern Qiangtang terrane yielded consistent palaeolatitudes of ~ 29.8–31.7°N, values similar to present-day latitudes^38,39^. These palaeolatitudes are significantly higher than those of the eastern Lhasa terrane. Huang et al.^39^ attribute this discrepancy to the giant right-lateral transpressional zone proposed by Dewey et al.^44^. However, an alternative explanation suggests that these Lower Cretaceous rocks of the Eastern Qiangtang terrane have been completely remagnetized^40^. Five Early Cretaceous palaeopoles from both volcanic and sedimentary rocks in the western Qiangtang terrane yield inconsistent palaeolatitudes, ranging from ~ 11.3°N^40^ to ~ 30.5°N^43^. Consequently, palaeomagnetic data suggest that the Lhasa-Qiangtang collision occurred at least by the Early Cretaceous^40^ or later than 115 Ma^42^. Factors that could account for the large palaeolatitude discrepancy observed in the western Qiangtang terrane are: lower palaeolatitudes from sedimentary rocks may be partially due to compaction-induced inclination shallowing^45,46^; and the absence of robust field tests to validate the reliability of palaeomagnetic data from volcanic rocks^42^. Therefore, more robust Early Cretaceous palaeomagnetic data from the Qiangtang terrane are needed to address these unresolved issues.

This study presents new, well-dated Early Cretaceous palaeomagnetic data from the Meiriqieco Formation rhyolites in the western Qiangtang terrane. These results significantly refine the Early Cretaceous palaeogeography of the western Qiangtang terrane and clarify the timing of the Lhasa-Qiangtang collision.

### Geological setting and samples

**Fig. 1:**
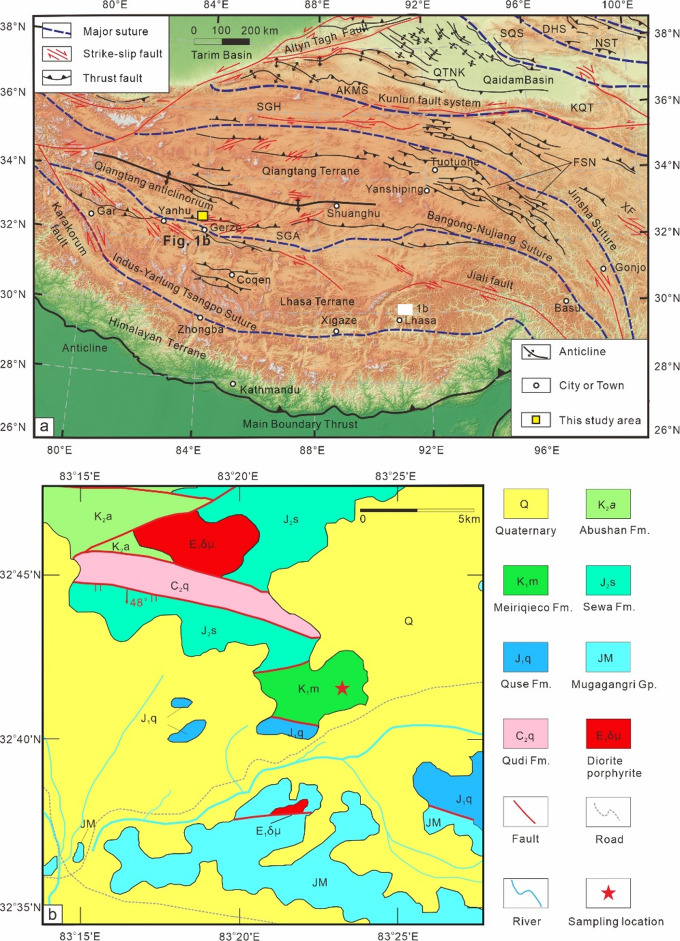
(**a**) Generalized tectonic map of the Tibetan Plateau and surrounding areas, modified after Wang et al.^10^. The map was prepared using QGIS software (https://www.qgis.org/en/site/) and the Global Digital Elevation Model V003 dataset (http://search.Earthdata.nasa.gov/search). Abbreviations: AKMS, Ayimaqin-KunlunMuztagh suture; DHS, Danghe Nan Shan suture; FSN, Fenghuo Shan–Nangqian fold-and-thrust belt; KQT, Kunlun-Qaidam terrane; QTNK, Qimen Tagh– North Kunlun thrust system; NQS, North Qilian Suture; NST, Nan Shan thrust belt; SGA, Shiquanhe-Gaize-Amdo thrust system; SGH, Songpan-Ganzi–Hoh Xil terrane; SQS, South Qilian suture. XF, Xianshuihe fault. (**b**) Simplified geologic map of the palaeomagnetic sampling sites in the Gerze area; Abbreviations: Fm., Formation; Gp., Group.

The Qiangtang terrane, situated between the Songpan-Ganzi and Lhasa terranes, is bounded by the Jinsha suture to the north and the Bangong-Nujiang suture to the south (Fig. 1a). The Longmu Cuo-Shuanghu suture zone subdivides the Qiangtang terrane into the Northern and Southern Qiangtang subterranes. Our study area is located in the Southern Qiangtang subterrane, about 80 km northwest of Gerze County, which exposes primarily Jurassic and Cretaceous strata, including the Quse (lower Jurassic), Sewa (middle Jurassic), Meiriqieco (lower Cretaceous), and Abushan (upper Cretaceous) formations (Fig. 1b). The Quse Formation consists mainly of quartz sandstone, siltstone, and mudstone. The Sewa Formation primarily comprises siltstone and mudstone. The Meiriqieco Formation is mainly composed of andesite, basaltic andesite, and rhyolite. The Abushan Formation primarily includes conglomerate and conglomeratic sandstone. The Meiriqieco Formation exhibits an angular unconformable contact with both the overlying Abushan Formation and the underlying Sewa Formation. The angular unconformity between the Lower Cretaceous Meiriqieco and Upper Cretaceous Abushan formations indicates that folding of the Meiriqieco Formation primarily occurred between the late Early Cretaceous and the Late Cretaceous.

We collected 306 palaeomagnetic samples from 31 palaeomagnetic sites along four adjacent hillocks in the field (32°41′30″N, 83°24′41″E) using a water-cooled portable drill. Samples were oriented using both magnetic and, when available, sun compasses. A discrepancy of less than 3° indicates minimal local magnetic disturbance. Bedding exhibits a dip of 82°–89° towards the southeast. Precise bedding attitudes were determined at the boundaries between distinct cooling margins or rhyolitic structures (Supplementary Fig. S1). Additionally, two fresh samples (BS32: 32°41′36″N, 83°22′47″E; BS3: 32°41′22″N, 83°23′20″E) were collected near our sampling sites for zircon U-Pb geochronology.

## Results

### Zircon U-Pb geochronologic results

**Fig. 2:**
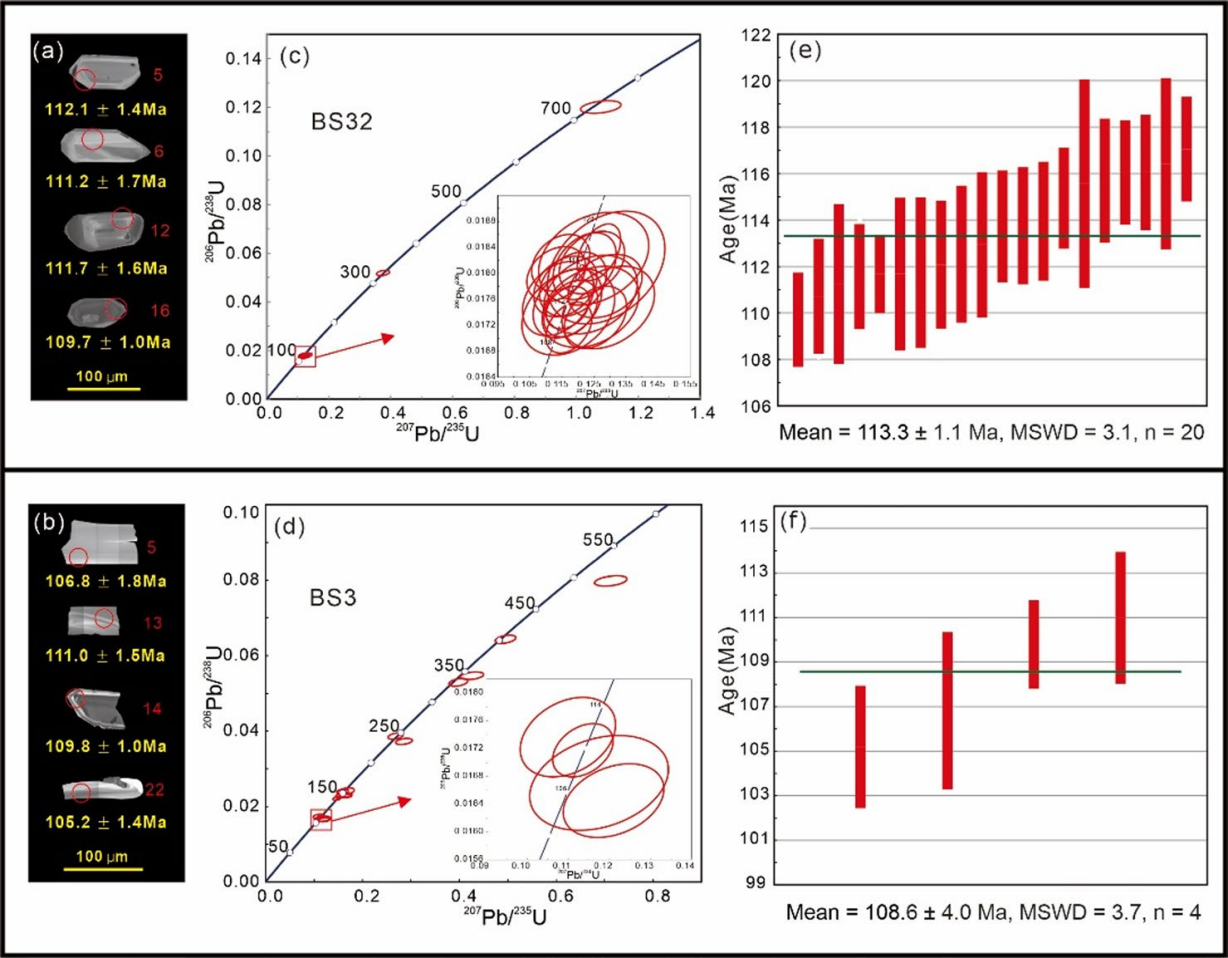
(**a**,** b**) Cathodoluminescence images of representative zircon grains and corresponding younger ^206^Pb/^238^U ages of the analyzed spots. (**c**,** d**) U–Pb concordia diagram of zircon grains. (**e**,** f**) Bar plot shows the weighted mean ^206^Pb/^238^U ages. Abbreviations: MSWD, mean square of weighted deviates; n, number of analyzed spots.

Zircon grains are euhedral to subhedral (~ 60–110 μm long, ~ 30–50 μm wide) and typically display clear oscillatory zoning (Fig. 2a, b). All zircons exhibit Th/U ratios exceeding 0.1 (Supplementary Table S1). Collectively, these observations support an interpretation of primary magmatic origin for the dated zircon grains. Laser Ablation Inductively Coupled Plasma Mass Spectrometry (LA-ICP-MS) U-Pb dating of zircons reveals a wide spectrum of ages (Fig. 2c, d), indicating that these zircons were derived from multiple sources. We interpret the weighted mean ^206^Pb/^238^U age obtained from the youngest population of zircons as the best approximation for the eruption age of the studied volcanic rocks. Zircon LA-ICP-MS U-Pb analyses of two Meiriqieco Formation rhyolite samples yielded youngest weighted mean ^206^Pb/^238^U ages of 113.3 ± 1.1 Ma (Fig. 2e) and 108.6 ± 4.0 Ma (Fig. 2f), respectively. Despite comprising only four analyses, the BS3 zircon U-Pb age (108.6 ± 4.0 Ma) is considered reliable due to analytical coherence within errors and stratigraphic consistency with published ages (see the Discussion section).

### Rock magnetic results

Isothermal Remanent Magnetization (IRM) acquisition curves for the Meiriqieco Formation rhyolite show a rapid increase below 100 mT, followed by a gradual increase up to 2.4 T (Supplementary Fig. S2a–c). Backfield demagnetization of the saturation IRM (SIRM) curves reveals a rapid decrease to zero at ~ 30–24 mT (Supplementary Fig. S2a–c). These data indicate the presence of both low- and high-coercivity magnetic minerals in the Meiriqieco Formation rhyolite.

IRM component analyses^47^ revealed three distinct components (Supplementary Fig. S2d–f): a soft component 1 with a B_1/2_ (the field at which half of the SIRM was acquired) ranging from ~ 27 to 34 mT, a hard component 2 with a B_1/2_ ranging from ~ 316 to 1230 mT, and a component 3 with a B_1/2_ ranging from ~ 4 to 794 mT. Component 1, interpreted as the primary magnetic carrier, contributed 78%–89% to the SIRM. Component 2, the subordinate magnetic carrier, contributed 5%–21% to the SIRM. Component 3 contributed 1%–9% to the SIRM and, while small, improved the data fit.

Thermal demagnetization of three-axis IRM (Supplementary Fig. S2g–i; Lowrie^48^ revealed unblocking temperatures of ~ 680 °C for the hard (2.4 T) and intermediate (0.4 T) components. The soft (0.12 T) component exhibited unblocking temperatures of ~ 680 °C with a slight inflection point observed around ~ 550 °C or ~ 580 °C. These results suggest that the soft component 1 is predominantly magnetite (possibly with minor Fe-substitution), while the hard component 2 is hematite.

### Petrographic results

Scanning electron microscopy (SEM) and energy dispersive spectrometry (EDS) of the Meiriqieco Formation rhyolites reveal abundant Ti-Fe and Fe oxides, typically occurring as irregular shapes distributed in silicate minerals (Supplementary Fig. S3). These Ti-Fe and Fe oxides, ranging in size from < 1 to > 100 μm, lack obvious oxidized grains rims. Some magnetic minerals exhibit structures with alternating light and dark stripes (Supplementary Fig. S3b), suggesting exsolution during rhyolite cooling^49^. Combined with rock magnetic results, our SEM-EDS analysis indicates that primary hematite and magnetite (with minor Fe-substitution) are the main magnetic phases.

### Palaeomagnetic results

**Fig. 3:**
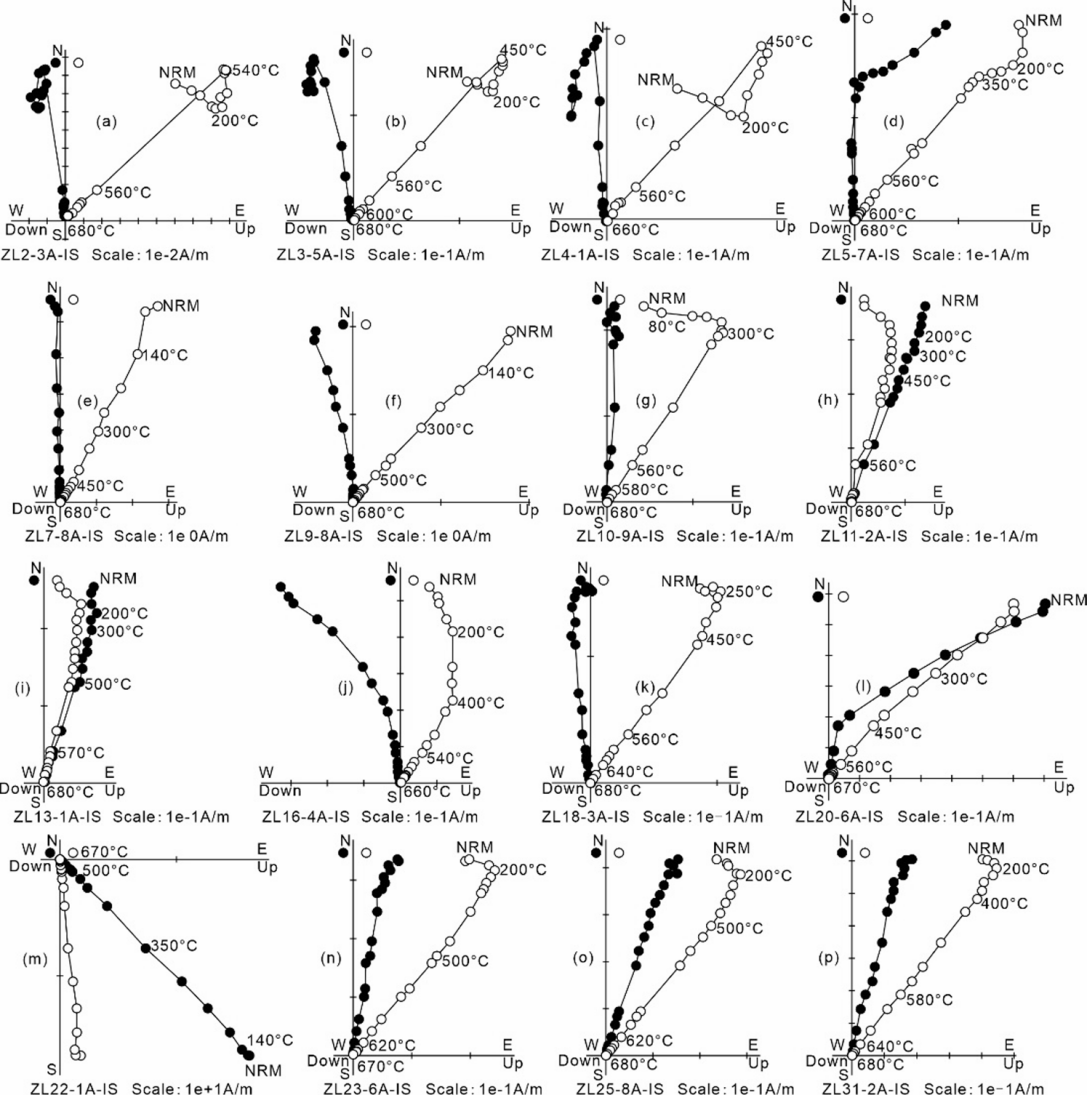
Typical demagnetization trajectories of representative specimens from the Lower Cretaceous Meiriqieco Formation rhyolite plotted in geographic coordinates. Solid (open) symbols represent projections onto the horizontal (vertical) plane. Abbreviations: NRM, natural remanent magnetization.

Two hundred and forty-one specimens underwent stepwise thermal demagnetization. Typical Zijderveld^50^ diagrams for the Meiriqieco Formation rhyolites are shown in Fig. 3. A low-temperature component (LTC) was isolated between room temperature and 300 °C for some specimens. The LTC’s mean direction in geographic coordinates (D = 2.4°, I = + 44.7°, k = 18.9, α_95_ = 4.0°; *n* = 71) closely aligns with the present local geomagnetic field direction (D = 1.3°, I = + 51.4°) and the geomagnetic dipole field (D = 0°, I = + 52.1°) (Supplementary Fig. S4), suggesting a recent overprint.

**Fig. 4:**
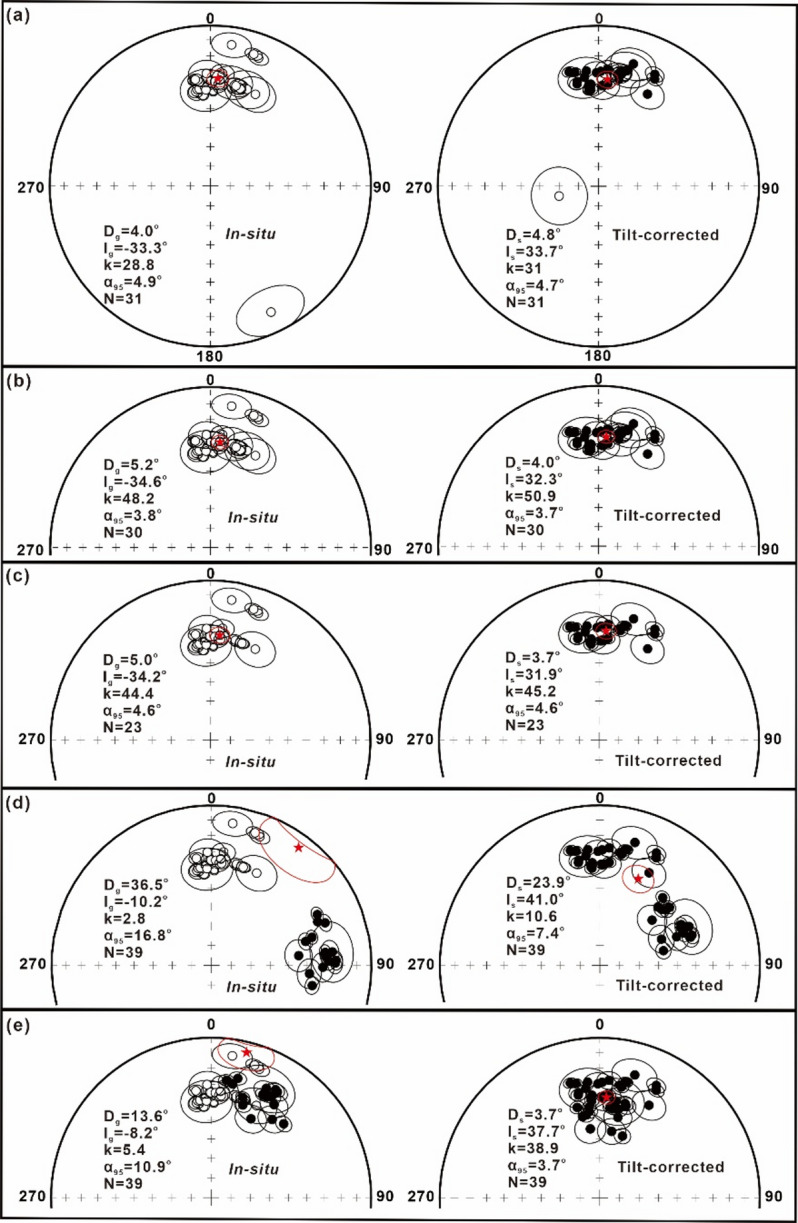
(**a**,** b**) Equal-area projections of site-mean directions from our study before and after tilt correction. The stars indicate the overall mean directions of (**a**) 31 and (**b**) 30 sites. (**c**) Equal-area projections of group-mean directions from our study before and after tilt correction. (**d**,** e**) Equal-area projections of site(group)-mean directions comparing our results with those of Cao et al.^42^, the latter rotated 57.2° counterclockwise to align with our data.

After removing the low-temperature component (LTC), a stable high-temperature component (HTC) was isolated from most specimens, primarily between 350 and 520 °C and 660–680 °C. The HTC directions, which include 197 of normal (Fig. 3a–l, n–p) and 9 of reverse polarities (Fig. 3m), are presented in Supplementary Table S2. The reversed HTC directions at site ZL22, which occur at the site level, indicate a geomagnetic reversal during the Cretaceous Normal Superchron (CNS, ca. 121–83.5 Ma)^51,52^. Notably, by synthesizing global records of polarity reversals across marine, volcanic, and terrestrial sediments within the CNS, Zhang et al.^53^ suggested the occurrence of at least five global and two single reversed-polarity events or clusters of events. Our geochronological data reveal an Early Cretaceous (ca. 113–109) age for the Meiriqieco Formation. This age corresponds to the Late Aptian–Early Albian reversed-polarity events or clusters identified by Zhang et al.^53^. The overall site-mean direction (overall mean A) is D_g_ = 4.0°, I_g_ = − 33.3°, k_g_ = 28.8, α_95_ = 4.9° (in situ) and D_s_ = 4.8°, I_s_ = + 33.7°, k_s_ = 31.0, α_95_ = 4.7° (after tilt correction), corresponding to a palaeopole at 75.6°N, 244.0°E, with A_95_ = 5.2° (*N* = 31) (Fig. 4a; Supplementary Table S3). Notably, the HTC directions from site ZL22 are identified as an outlier by the Jackknife test^55^, potentially representing an excursional or transitional direction. After excluding site ZL22, the remaining 30 sites yield a site-mean direction (overall mean B) of D_g_ = 5.2°, I_g_ = − 34.6°, k_g_ = 48.2, α_95_ = 3.8° (in situ) and D_s_ = 4.0°, I_s_ = + 32.3°, k_s_ = 50.9, α_95_ = 3.7° (after tilt correction), corresponding to a palaeopole at 74.5°N, 249.5°E with A_95_ = 3.9° (Fig. 4b; Supplementary Table S3). Fold tests yield insignificant results given minimal variation in bedding attitude across sampled Meiriqieco Formation rhyolites.

Because successive lava flows can be emplaced more rapidly than the geomagnetic field changes, we calculated group-mean directions (instead of site-mean directions) for stratigraphically successive sites sharing a common palaeomagnetic direction. Thirty of 31 palaeomagnetic sites were grouped into 23 independent directional groups, which yield a group-mean direction (overall mean C) of D_g_ = 5.0°, I_g_ = − 34.2°, k_g_ = 44.4, α_95_ = 4.6° (in situ) and D_s_ = 3.7°, I_s_ = + 31.9°, k_s_ = 45.2, α_95_ = 4.6° (after tilt correction), corresponding to a palaeopole at 74.3°N, 250.5°E with A_95_ = 4.7° (Fig. 4c; Supplementary Table S3). The group-mean direction closely matches the site-mean direction.

We followed the established method of Deenen et al.^56^ to determine if the palaeomagnetic data from the Gerze area have undergone averaging of palaeosecular variation. Given an A_95_ value of 4.7° calculated from the VGPs of 23 volcanic groups, which aligns with the predicted N-dependent A_95min_/A_95max_ ratio of 3.4°/11.4°, we conclude that the group-mean direction of the Meiriqieco Formation rhyolites has averaged geomagnetic palaeosecular variation.

## Discussion

Interpreting palaeomagnetic data requires reliable age control for the sampled strata. Yet, the intricate geologic history of the Tibetan Plateau presents a significant challenge, often resulting in inaccurate age assignments for numerous stratigraphic units^26,29,30,57–61^. In the 1:250 000 Wuma regional geologic map (I44C004004, 2006), the sampled strata are assigned to the Lower Cretaceous Meiriqieco Formation. However, zircon LA-ICP-MS U-Pb dating by Dong et al.^62^ yielded an age of 150.5 ± 0.6 Ma, and assigned the sampled strata to the Upper Jurassic Duiwangshan Formation. Notably, their analysis spots were located near the zircon cores. Targeting the rims of zircons, our new LA-ICP-MS U-Pb dating indicates that the sampled strata erupted during ca.113 to 109 Ma. These updated chronologic results agree with ages of ca. 117–116 Ma^42^ and ca. 113–108 Ma^63^ for the Meiriqieco Formation volcanic rocks in adjacent regions. Therefore, we conclude that the sampled lavas of the Meiriqieco Formation were erupted during the Early Cretaceous.

Because the Lhasa and Qiangtang terranes collided primarily in a north-south direction^10,20^, their palaeolatitudinal variations at a common reference point can directly determine the timing and location of their collision. Recently, Wang et al.^10^ synthesized robust Jurassic palaeomagnetic data from the eastern Lhasa (east of 87°E)^64–67^ and Qiangtang^20,68^ terranes. Their results indicate that during the Middle Jurassic (~ 170 Ma), the eastern Lhasa and Qiangtang terranes were located at palaeolatitudes of 14.4 ± 5.7°N and 19.2 ± 2.5°N for the reference point (29.7°N, 91.3°E), respectively. Assuming a constant northward velocity of ~ 24.3 cm/yr between ~ 180 and 170 Ma, they concluded that the eastern Lhasa-Qiangtang terranes collided at ~ 168 Ma. This collisional age, which strongly supports existing geological estimates^10^, is therefore deemed credible. Considering (1) the potential for a diachronous Lhasa-Qiangtang collision^10,20^ and (2) the lack of reliable Late Jurassic palaeomagnetic data for the Qiangtang terrane, we focused our analysis on the western collision zone (west of 87°E) to determine if the Lhasa and Qiangtang terranes overlapped during the Early Cretaceous.

Table 1 lists available Early Cretaceous palaeomagnetic datasets from the western Lhasa and Qiangtang terranes, which were evaluated using the updated R criteria^69^. In this study, palaeomagnetic data must exhibit either a robust field test (e.g., positive fold or conglomerate tests) or a positive reversal test, in addition to meeting all other five R criteria. Palaeolatitudes were calculated using a reference point of (32.7°N, 83.4°E), located within our study area.

**Table 1:** Summary of early cretaceous Palaeopoles from the Western Lhasa and Qiangtang terranes.

ID	lithology	Area	Slat	Slon	Age	Plat	Plon	A_95_ (dp/dm)	Palaeolat	*n*/*N*	Criterion (Q)	References
			(°*N*)	(°E)	(Ma)	(°*N*)	(°E)	(°)	(°*N*)			
Early Cretaceous palaeomagnetic results from the western Qiangtang terrane												
LM	RB	Longmucuo	34.5	80.4	Aptian- Albian	64.4	231.3	12.8	10.3 ± 12.8	41/4	123F5 × 7 (6)	^40^
AK	RB	Aksaichin	35.0	79.7	Aptian- Albian	66.3	256.5	6.6	9.1 ± 6.6	44/7	123F5 × 7 (6)	^40^
MR1	Volc	Gerze	32.9	83.5	~ 120 − 115	37.9	162.2	5.1	27.4 ± 5.1	127/16	123 × 5 × 7 (5)	^42^
MR2	Volc	Gerze	32.7	83.4	~ 113 − 109	74.3	250.5	4.7	17.3 ± 4.7	197/23	123 × 5 × 7 (5)	This study
MR1 + 2	Volc	Gerze	-	-	~ 120 − 109	78.3	246.5	3.7	21.5 ± 3.7	324/39	123F5 × 7 (6)	This study
GZ	Volc	Gerze	32.5	84.3	~ 111 − 104	79.3	339.8	5.7	29.6 ± 5.7	91/14	123F5D7 (7)	^41^
LJ	Volc	Lumajiangdong	34.2	81.4	~ 110 − 100	15.4	144.7	5.0	32.2 ± 5.0	281/42	123 F*5 × 7 (7)	^43^
Early Cretaceous palaeomagnetic results from the western Lhasa terrane												
QS	Volc	Yanhu	32.3	82.6	~ 132 − 120	61.4	192.9	2.1	19.9 ± 2.1	444/51	123F5D7 (7)	^25^
ZN	Volc	Cuoqin	31.4	85.1	~ 131 − 110	58.2	341.9	4.6	21.8 ± 4.6	162/18	123F5R7 (7)	^24^
DZ	Volc	Cuoqin	31.1	84.4	~ 121 − 117	70.5	292.9	7.4	15.4 ± 7.4	116/12	123 F*5D7 (7)	^26^
WMa	Volc + RB	Gerze	32.4	83.4	~ 120 − 106	38.7	159.6	1.7/2.6	29.6 ± 1.7	198/24	123F5D7 (7)	^29^
WMb	RB	Gerze	32.4	83.4	~ 120 − 106	75.2	278.1	1.5/2.6	18.3 ± 1.5	255/32	123F5D7 (7)	^29^
SQ	Volc + Lim	Shiquanhe	32.2	80.4	~ 116 − 113	67.7	322.0	3.8	19.5 ± 3.8	242/29	123F5 × 7 (6)	^27^

*Notes*: ID, palaeopoles abbreviation used in the plot and text; RB, red beds; volc, volcanic rocks; Lim, limestones; Slat (Slon), latitude (longitude) of sites; Plat (Plon), latitude (longitude) of poles; A_95_, the radius that the mean pole lies within 95% confidence; dp/dm, semi-axes of elliptical error of the pole at a probability of 95%; Palaeolat, palaeolatitude calculated for the reference point at (32.7ºN, 83.4ºE); n/N, number of samples or sites used to calculate Fisher mean; Criteria (Q), data quality criteria (number of criteria met) after Meert et al.^69^ (1, well determined rock age and a presumption that magnetization is the same age; 2, techniques and statistical analysis; 3, evaluation of remanence carriers; 4, field tests that constrain age of magnetization; 5, structural control and tectonic coherence with craton or block involved; inclination shallowing assessed in clastic sedimentary rocks; 6, the presence of reversals; 7, no resemblance to palaeopoles of younger ages [by more than a period]; F, positive fold test; F^∗^, positive fold test with additional data; R, positive reversal test; D, dual-polarity; “X”, failed to meet this criterion).

Our new palaeopole yields a palaeolatitude of 17.3° ± 4.7°N at ca. 113–109 Ma for the reference point (32.7°N, 83.4°E). Furthermore, Cao et al.^42^ reported reliable Early Cretaceous palaeomagnetic results from the Meiriqieco Formation volcanic rocks in the Gerze area. These data, from a similar time frame and nearby locations, provide an opportunity to further assess the reliability of the Meiriqieco Formation volcanic data through field tests. Merging our HTCs with data from Cao et al.^42^ yields a site-mean direction (Overall mean D) of D_g_ = 36.5°, I_g_ = − 10.2°, k_g_ = 2.8, α_95_ = 16.8° (in situ) and D_s_ = 23.9°, I_s_ = + 41.0°, k_s_ = 10.6, α_95_ = 7.4° (after tilt correction), corresponding to a palaeopole at 65.9°N, 188.0°E, with A_95_ = 8.6° (Supplementary Table S3, Fig. 4d). This combined direction passes the McElhinny^70^ fold test at the 95% and 99% confidence levels: k_s_/k_g_ = 3.73 > F (76, 76) at 5% point = 1.46 and at 1% point = 1.71. This combined direction also passes the McFadden^54^ fold test at the 95% and 99% confidence levels: the calculated values are ξ_(2)in situ_ = 32.94 in geographic coordinates and ξ_(2)tilt corrected_ = 0.17 after tilt correction, while the critical values are ξ_C_ = 7.26 at 95% confidence level, and ξ_C_ = 10.27 at 99% confidence level. Notably, the declinations presented in this study and those of Cao et al.^42^ exhibit significant discrepancies (Fig. 4d), plausibly resulting from regional faulting that induced local vertical-axis rotation of the sampling sites. Therefore, an inclination-only fold test^71^ was used to analyze palaeomagnetic data from sampling sections possibly affected by local rotations. The inclination-only fold test yielded a ks/kg ratio of 21.3 (43.4/2.04), exceeding both the 95% (1.46) and 99% (1.71) confidence limits, indicating a positive result. Secondly, following a 57.2° counterclockwise rotation of the Cao et al.^42^ site-mean directions, the combined HTCs from the Meiriqieco Formation yield a site-mean direction (Overall mean E) of D_g_ = 13.6°, I_g_ = − 8.2°, k_g_ = 5.4, α_95_ = 10.9° (in situ) and D_s_ = 3.7°, I_s_ = + 37.7°, k_s_ = 38.9, α_95_ = 3.7° (after tilt correction), corresponding to a palaeopole at 78.3°N, 246.5°E, with A_95_ = 3.7° (Supplementary Table S3, Fig. 4e). This direction passes the McElhinny^70^ fold test at both the 95% and 99% confidence levels: k_s_/k_g_ = 7.23 > F (76, 76) at 5% point = 1.46 and at 1% point = 1.71. Therefore, we interpret the HTCs from the Meiriqieco Formation volcanic rocks as pre-folding and likely primary in origin.

**Fig. 5:**
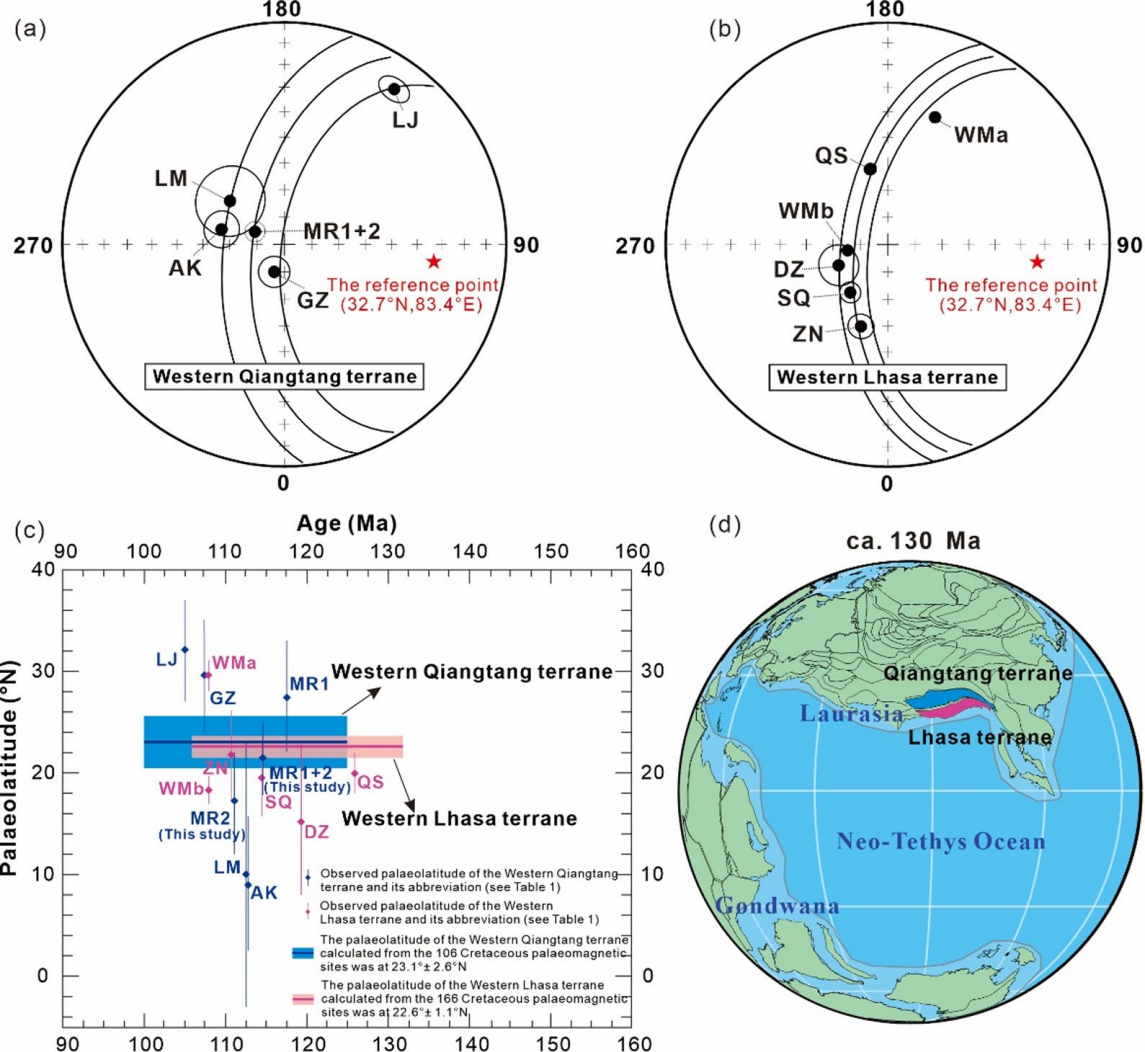
(**a**,** b**) Equal-area projections showing the Early Cretaceous palaeomagnetic poles obtained from the western Qiangtang and Lhasa terranes. (**c**) Palaeolatitude plots for the western Qiangtang and Lhasa terrane. The shaded areas and vertical bars show the errors of the palaeolatitude. Expected and observed palaeolatitudes were calculated for a reference point at 32.7°N, 83.4°E. (**d**) Palaeogeographic reconstructions at ca.130 Ma based on this study and Torsvik and Cocks^84^ using Gplates (Müller et al.^85^ https://portal.gplates.org/#apps-anchor).

Our new palaeomagnetic data, combined with the data of Cao et al.^42^, indicate that the western Qiangtang terrane was located at 21.5° ± 3.7°N (Table 1). Song et al.^43^ reported an Early Cretaceous (ca. 110–100 Ma) palaeopole (15.4°N, 144.7°E, A_95_ = 5.0°) from volcanic rocks in the far western Qiangtang terrane, indicating a palaeolatitude of ∼32.2 ± 5.0°N. Although their samples were collected from strata exposed in a monoclinic, their palaeomagnetic data, combined with that of Chen et al.^40^, passed the fold test. The other three Early Cretaceous palaeopoles from the western Qiangtang terrane, which meet our quality criteria, indicate palaeolatitudes of 10.3° ± 12.8°N^40^, 9.1° ± 6.6°N^40^, and 29.6° ± 5.7°N^41^, respectively (Table 1). Despite the palaeolatitude of the western Qiangtang terrane underwent certain changes during ~ 120–100 Ma, the corresponding palaeopoles are statistically indistinguishable within 95% confidence limits and aligned along a small circle centered on (32.7°N, 83.4°E) (Fig. 5a). Therefore, we calculated the colatitude, and hence palaeolatitude, using the method of Mardia and Gadsden^72^ and the procedure of Cogné^55^.A colatitude of 69.4° ± 13.3°N, derived from the fitted small circle passing through the five selected palaeopoles, translates to a palaeolatitude of 20.6° ± 13.3°N for the western Qiangtang terrane. We also calculated the inclination-only mean following the method of Arason and Levi^73^, and then determined its corresponding palaeolatitude. This analysis, based on 106 palaeomagnetic sites, yielded an inclination-only mean of + 43.1° ± 3.4° (Supplementary Table S4) and a corresponding palaeolatitude of 25.1° ± 2.6°N. The results from both methods are consistent. Therefore, we adopt a mean palaeolatitude of 25.1° ± 2.6°N as the Early Cretaceous palaeolatitude for the western Qiangtang terrane.

The available Early Cretaceous palaeopoles from the western Lhasa terrane, all meeting our quality criteria (Table 1), indicate palaeolatitudes of 19.9° ± 2.1°N^25^, 21.8° ± 4.6°N^24^, 15.4° ± 7.4°N^26^, 29.6° ± 1.7°N^29^, 18.3° ± 1.5°N^29^, and 19.5° ± 3.8°N^27^. Because these palaeopoles are statistically indistinguishable within 95% confidence limits and align along a small circle centered on the reference point (32.7°N, 83.4°E) (Fig. 5b), we calculated the colatitude using the method mentioned above to obtain the corresponding palaeolatitude. The fitted small circle, which passes through the six selected palaeopoles, yields a colatitude of 69.3° ± 5.1°N, indicating a palaeolatitude of 20.7° ± 5.1°N for the western Lhasa terrane. Following the method of Arason and Levi^73^, we obtained an inclination-only mean of + 36.9° ± 1.6° (Supplementary Table S4) and a corresponding palaeolatitude of 20.6° ± 1.1°N from 166 Early Cretaceous palaeomagnetic sites. Given the agreement with results from the method of Mardia and Gadsden^72^, we adopt 20.6° ± 1.1°N as the preferred Early Cretaceous palaeolatitude for the western Lhasa terrane. In light of potential shortening within a pre-collisional forearc basin and 120–250 km of Cenozoic shortening accommodated by the Shiquanhe-Gaize-Amdo thrust system^3^, we follow the approach of Lippert et al.^74^ and add 2° of latitude to both the southern Qiangtang and northern Lhasa terrane margins. Accordingly, we derive a palaeolatitude of 23.1° ± 2.6°N for the western Qiangtang’s southern margin, and 22.6° ± 1.1°N for the Lhasa terrane’s northern margin. The statistical indistinguishability of these palaeolatitudes suggests that the western Lhasa and Qiangtang terranes collided before the Early Cretaceous (ca. 132–100 Ma; Fig. 5c, d).

This collisional age is compatible with results from other geologic studies, such as: (1) a comprehensive analysis of ophiolites, metamorphism, magmatism, lithostratigraphy, and tectonism in the Gerze area suggests that the Lhasa-Qiangtang collision occurred at ~ 150–130 Ma^11^; (2) structural mapping and detrital zircon geochronology from the Domar fold-thrust belt in the western Qiangtang terrane by Raterman et al.^15^ show that upper crustal shortening was likely driven by the convergence between the Lhasa and Qiangtang terranes during the Late Jurassic to Early Cretaceous; (3) detrital zircon analysis suggests the 140–130 Ma magmatic gap in the Gerze area reflects the Lhasa-Qiangtang collision^75^ (4) the integrated published apatite fission track and (U–Th)/He thermochronometer data with new zircon (U–Th)/He data from the western Qiangtang terrane, leading Zhao et al.^76^ to suggest latest Jurassic–earliest Cretaceous exhumation in the area, linked to Bangong Ocean closure and subsequent crustal shortening and thickening.

## Conclusions

We present new geochronologic and palaeomagnetic results from the Lower Cretaceous Meiriqieco Formation rhyolite lavas in the western Qiangtang terrane. Integrating these data with existing Early Cretaceous palaeomagnetic data from the western Lhasa and Qiangtang terranes, we draw the following conclusions: (1) the Meiriqieco Formation rhyolites in the Gerze area erupted at ~ 113–109 Ma, revising previous Late Jurassic age estimates; (2) the western Qiangtang terrane was located at ~ 23.1°N during the Early Cretaceous; and (3) the western Lhasa and Qiangtang terranes had already converged by the Early Cretaceous (between about 132 and 100 Ma).

## Methods

### Palaeomagnetic measurements and analyses

Oriented core samples (2.5 cm diameter) were cut into 2.2-cm-long specimens for palaeomagnetic and rock-magnetic analyses. Remanent magnetization was measured using a JR-6 A spinner magnetometer, and demagnetization was carried out using an ASC-TD 48 furnace (internal residual field < 10 nT) with temperature steps of 50 °C in the low-temperature range (typically below 400 °C) and 10–30 °C in the h^ig^h-temperature range. The furnace and magnetometers were housed in a magnetically shielded room (< 300 nT) at the Palaeomagnetism and Environmental Magnetism Laboratory (PMEML), China University of Geosciences, Beijing (CUGB). Remanent magnetization directions of all specimens were determined via principal component analyses^77^. Fisher^78^ statistics were subsequently used to calculate site-mean directions. Computational packages developed by Enkin^79^ and Cogné^55^ were used for all palaeomagnetic data analyses.

### Rock magnetic experiments

We characterized magnetic mineralogy using: (1) IRM acquisition and back-field demagnetization; and (2) thermal demagnetization of three-axis IRM^48^. An ASC IM10-30 pulse magnetizer imparted the IRM, while an ASCTD 48 furnace (internal field < 10 nT) performed thermal demagnetization of the three-axis IRM (fields of 2.4, 0.4, 0.12 T along orthogonal axes). A JR-6 A spinner magnetometer measured the IRM acquisition and back-field demagnetization data, along with the three-axis IRM thermal demagnetization. Following Kruiver et al.^47^, we analyzed IRM acquisition curves using the cumulative log-Gaussian approach to assess the relative content of magnetic minerals. All experiments were performed at the PMEML, CUGB.

### Petrographic analyses

Polished thin sections of representative samples were prepared for SEM observations and EDS analyses. SEM observations were performed at the Electron Microprobe Analyses and Scanning Electron Microscope Laboratory (Institute of Geology and Geophysics, Chinese Academy of Sciences) with a 15 kV acceleration voltage and 8–9 mm working distance. Samples were carbon-coated before SEM to improve image quality. EDS analyses provided composition information.

### Zircon U-Pb experiments

Zircon grains from two block samples were selected at the Langfang Gengxin Geological Service Company. CL imaging and zircon target preparation were performed at Beijing Gaonianlinghang Technology Limited Company. U-Pb analyses were conducted using LA-ICP-MS at the Mineral Laser Microprobe Analysis Laboratory, CUGB. Isotope ratios were calibrated against the NIST612 standard as an external standard^80^. Common lead correction followed the procedure outlined by Andersen^81^. Age data processing and analysis were carried out using the ICPMS DataCal^82^ and Isoplot^83^ software packages.

## Supplementary Information

Below is the link to the electronic supplementary material.

Supplementary Material 1

## Data Availability

The datasets generated during and/or analysed during the current study are available from the corresponding author on reasonable request.
