# Supplementary material for: Palaeomagnetic and geochronologic results from lower cretaceous volcanics of the western Qiangtang terrane and implications for the Lhasa-Qiangtang collision
Source: Sci Rep. 2025 Nov 27;15:45551. doi: 10.1038/s41598-025-30138-7 (PMC12749633; doi:10.1038/s41598-025-30138-7)
Supplement: Supplementary file 1 — Supplementary Material 1 [file 41598_2025_30138_MOESM1_ESM.docx]

**Supplementary Information**

**Palaeomagnetic and Geochronologic results from Lower Cretaceous volcanics of the western Qiangtang terrane and Implications for the Lhasa-Qiangtang Collision**

Yabo Zhang^1^, Weiwei Bian^1,*^, Jiahui Ma^1,2^, Suo Wang^1,3^, Xianwei Jiao^4^, Jiacheng Liang^1^, Siqi Wang^1^, Xiaolin Li^1^, Jikai Ding^1^, Hanqing Zhao^1^, Haiyan Li^1^, Huaichun Wu^1^, Yiming Ma^5^, and Tianshui Yang^1,*^

^1^ State Key Laboratory of Geomicrobiology and Environmental Changes, China University of Geosciences, Beijing, China.

^2^ State Key Laboratory of Lithospheric and Environmental Coevolution, Institute of Geology and Geophysics, Chinese Academy of Sciences, Beijing, China

^3^ Key Lab of Submarine Geosciences and Prospecting Techniques, Ocean University of China, Qingdao, China

^4^ Beijing Institute of Geological Survey, Beijing, China

^5^ School of Earth Sciences, China University of Geosciences, Wuhan, Hubei, China

*Corresponding authors: Weiwei Bian (email: [bianww@cugb.edu.cn](mailto:bianww@cugb.edu.cn)) and Tianshui Yang (email: [yangtsh@cugb.edu.cn](mailto:yangtsh@cugb.edu.cn))


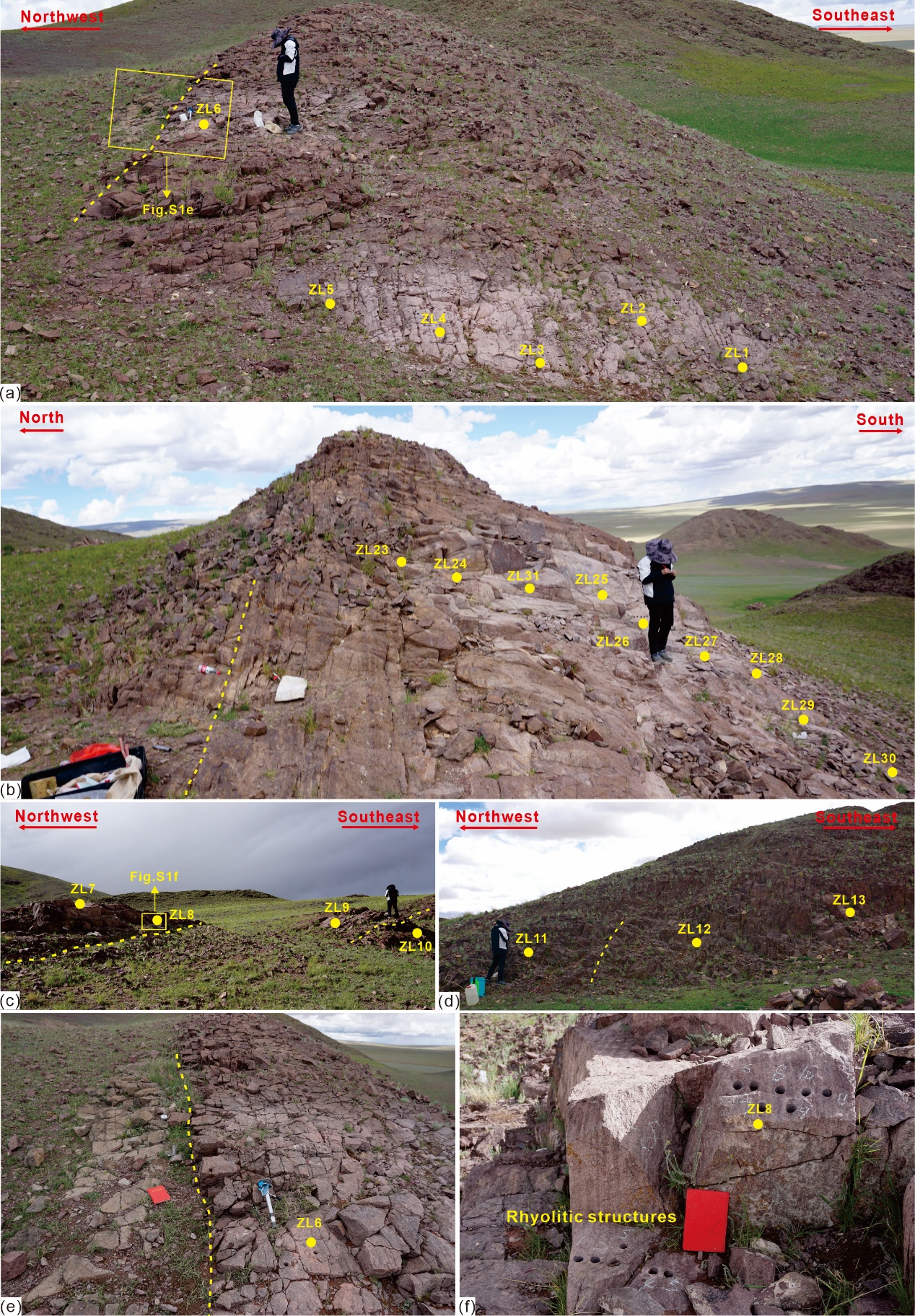
**Supplementary Figure S1.** Photographs showing the field outcrops of sampling sections and sites of the Meiriqieco Formation rhyolite in the western Qiangtang Terrane.


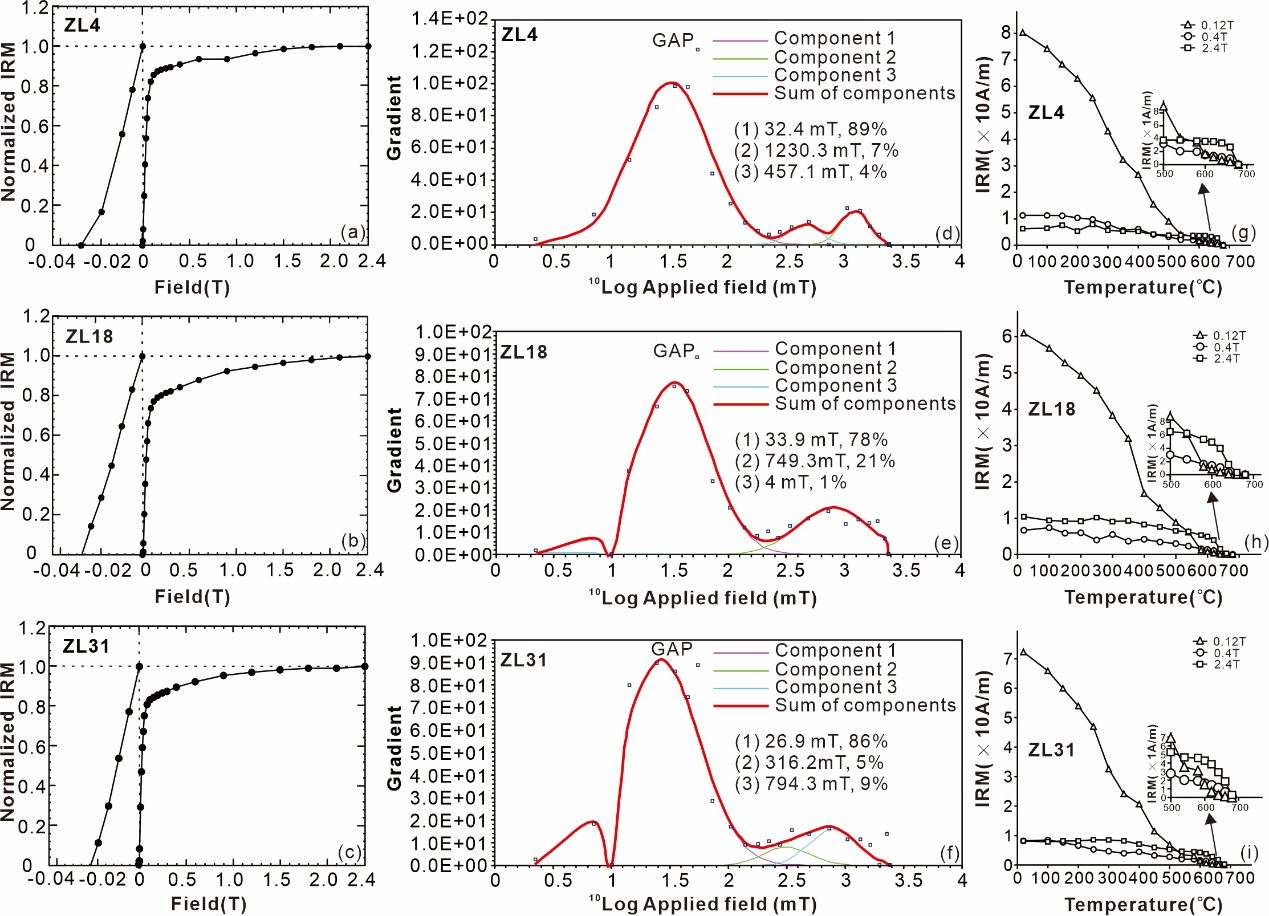
**Supplementary Figure S2.** (a–c) Isothermal remanent magnetization (IRM) acquisition curves and back-field demagnetization of saturation IRM curves. (d–f) Component analysis of coercivity distributions (Kruiver et al_._^47^). (g–i) Thermal demagnetization of three-axis IRM curves. Abbreviations: GAP, gradient acquisition plot.


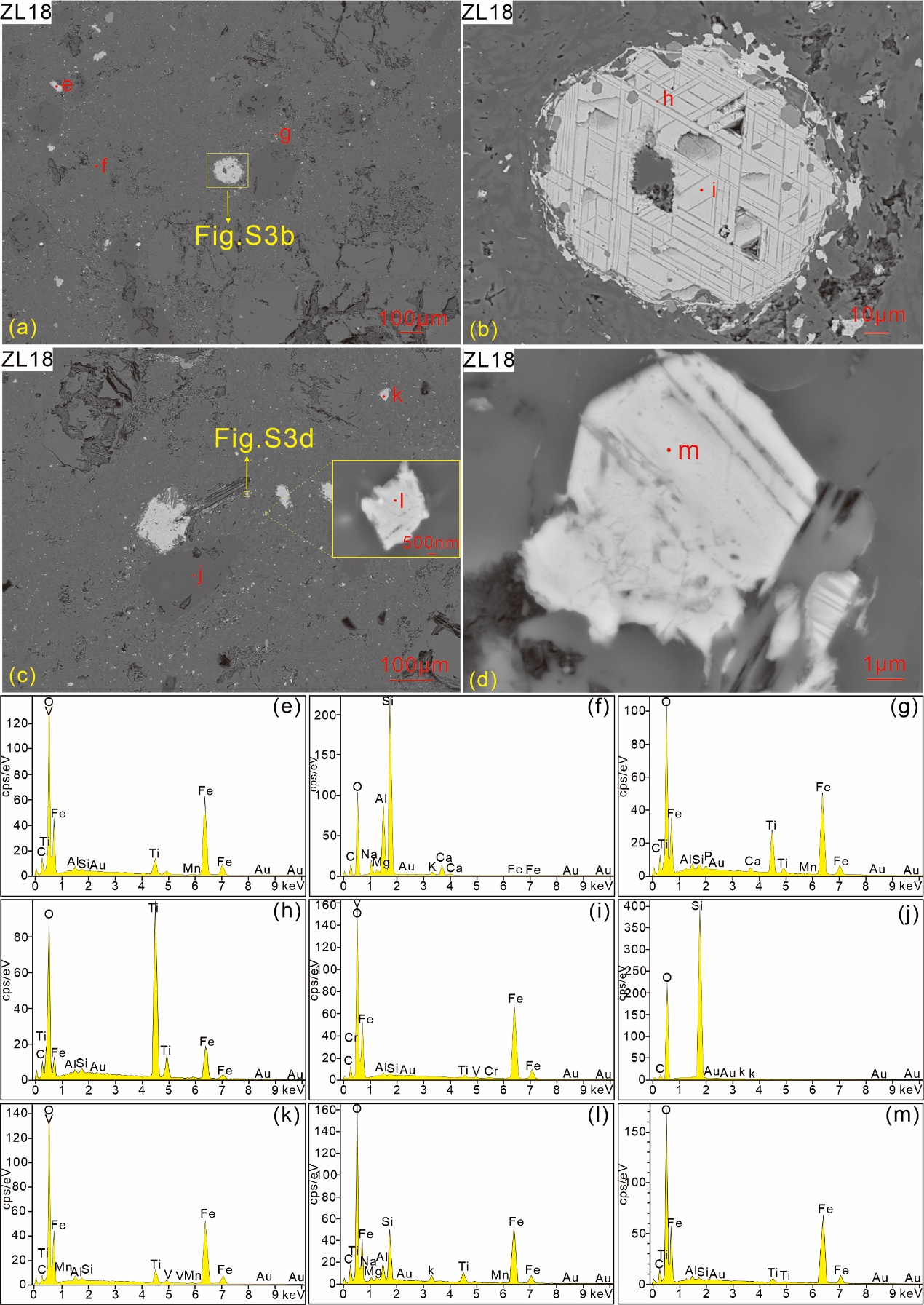
**Supplementary Figure S3.** (a–d) Scanning electron microscopy (SEM) observations and (e–m) the results of the energy dispersive spectrometry (EDS) analyses of representative samples of the Early Cretaceous Meiriqieco Formation rhyolite. The red spots show the EDS analytical points.


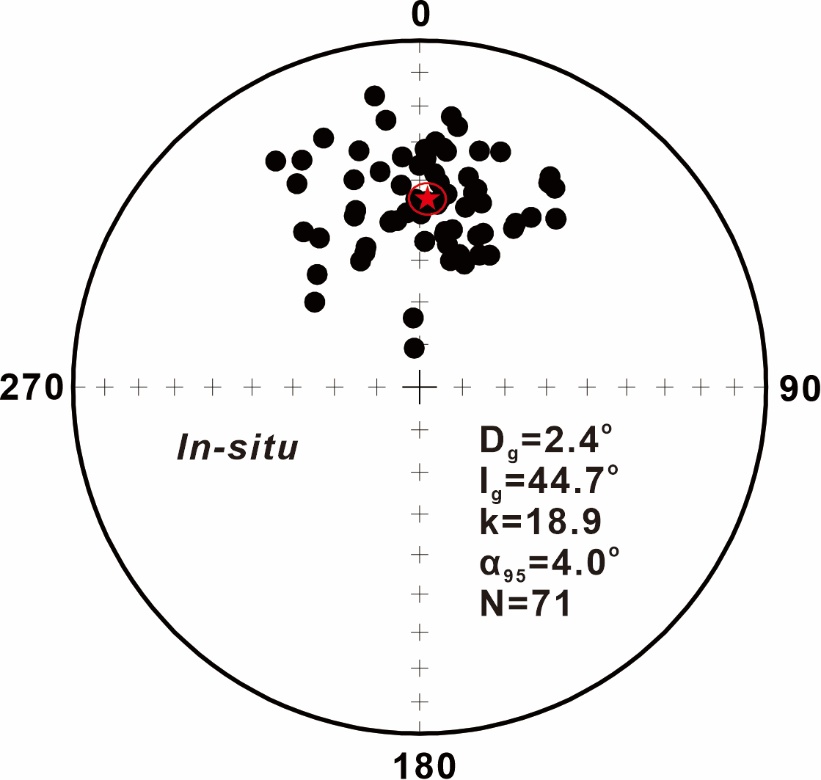


**Supplementary Figure S4.** Equal-area projection of the site-mean directions of the low-temperature component (LTC) of the Meiriqieco Formation rhyolite. The red star indicates the overall mean direction.

**Supplementary Table S1.** Summary of zircon U-Pb data of the sampling volcanic rocks.

| Spot | Total Pb | ^232^Th | ^238^U | Th/U | ^207^Pb/^235^U | | ^206^Pb/^238^U | | ^207^Pb/^235^U | | ^206^Pb/^238^U | | Concordance |
| --- | --- | --- | --- | --- | --- | --- | --- | --- | --- | --- | --- | --- | --- |
|  | ppm | ppm | ppm |  | Ratio | 1sigma | Ratio | 1sigma | Age (Ma) | 1sigma | Age (Ma) | 1sigma |  |
| **BS32** |  |  |  |  |  |  |  |  |  |  |  |  |  |
| BS32-16 | 17.6 | 705 | 905 | 0.78 | 0.1172 | 0.0029 | 0.0172 | 0.0002 | 112.5 | 2.6649 | 109.7 | 1.0147 | 97% |
| BS32-23 | 9.2 | 428 | 453 | 0.94 | 0.1255 | 0.0040 | 0.0173 | 0.0002 | 120.0 | 3.5927 | 110.7 | 1.2410 | 91% |
| BS32-06 | 3.7 | 141 | 183 | 0.77 | 0.1170 | 0.0059 | 0.0174 | 0.0003 | 112.3 | 5.3779 | 111.2 | 1.7233 | 99% |
| BS32-03 | 10.3 | 510 | 475 | 1.07 | 0.1177 | 0.0034 | 0.0175 | 0.0002 | 113.0 | 3.1215 | 111.6 | 1.1319 | 98% |
| BS32-20 | 24.8 | 951 | 1278 | 0.74 | 0.1168 | 0.0027 | 0.0175 | 0.0001 | 112.2 | 2.5011 | 111.7 | 0.8460 | 99% |
| BS32-21 | 5.0 | 230 | 241 | 0.95 | 0.1295 | 0.0059 | 0.0175 | 0.0003 | 123.6 | 5.3105 | 111.7 | 1.6437 | 89% |
| BS32-12 | 4.9 | 223 | 241 | 0.93 | 0.1239 | 0.0062 | 0.0175 | 0.0003 | 118.6 | 5.5762 | 111.7 | 1.6161 | 94% |
| BS32-05 | 8.1 | 395 | 382 | 1.04 | 0.1167 | 0.0044 | 0.0175 | 0.0002 | 112.1 | 3.9713 | 112.1 | 1.3785 | 99% |
| BS32-19 | 6.1 | 264 | 305 | 0.87 | 0.1138 | 0.0045 | 0.0176 | 0.0002 | 109.5 | 4.1426 | 112.5 | 1.4753 | 97% |
| BS32-14 | 4.9 | 229 | 226 | 1.01 | 0.1240 | 0.0060 | 0.0177 | 0.0002 | 118.6 | 5.3878 | 112.9 | 1.5655 | 95% |
| BS32-22 | 10.2 | 567 | 464 | 1.22 | 0.1161 | 0.0040 | 0.0178 | 0.0002 | 111.5 | 3.6788 | 113.7 | 1.2062 | 98% |
| BS32-18 | 7.6 | 286 | 378 | 0.76 | 0.1285 | 0.0047 | 0.0178 | 0.0002 | 122.7 | 4.2436 | 113.8 | 1.2610 | 92% |
| BS32-01 | 9.4 | 467 | 428 | 1.09 | 0.1184 | 0.0043 | 0.0178 | 0.0002 | 113.7 | 3.8813 | 113.9 | 1.2819 | 99% |
| BS32-08 | 12.0 | 621 | 527 | 1.18 | 0.1132 | 0.0037 | 0.0180 | 0.0002 | 108.9 | 3.4145 | 114.9 | 1.0859 | 94% |
| BS32-04 | 2.6 | 88 | 126 | 0.70 | 0.1288 | 0.0075 | 0.0181 | 0.0004 | 123.0 | 6.7482 | 115.6 | 2.2465 | 93% |
| BS32-02 | 9.9 | 491 | 443 | 1.11 | 0.1221 | 0.0040 | 0.0181 | 0.0002 | 117.0 | 3.6555 | 115.7 | 1.3310 | 98% |
| BS32-13 | 14.1 | 496 | 700 | 0.71 | 0.1146 | 0.0037 | 0.0182 | 0.0002 | 110.2 | 3.3712 | 116.0 | 1.1231 | 94% |
| BS32-15 | 9.9 | 436 | 469 | 0.93 | 0.1244 | 0.0042 | 0.0182 | 0.0002 | 119.0 | 3.7690 | 116.0 | 1.2446 | 97% |
| BS32-11 | 3.8 | 173 | 174 | 1.00 | 0.1249 | 0.0070 | 0.0182 | 0.0003 | 119.5 | 6.3409 | 116.4 | 1.8436 | 97% |
| BS32-17 | 29.4 | 707 | 1549 | 0.46 | 0.1266 | 0.0028 | 0.0183 | 0.0002 | 121.0 | 2.4973 | 117.1 | 1.1263 | 96% |
| BS32-10* | 11.3 | 368 | 379 | 0.97 | 0.2797 | 0.0117 | 0.0232 | 0.0003 | 250.4 | 9.3064 | 148.1 | 1.7282 | 48% |
| BS32-09 | 18.9 | 135 | 353 | 0.38 | 0.3759 | 0.0087 | 0.0519 | 0.0005 | 324.0 | 6.4061 | 326.0 | 2.7973 | 99% |
| BS32-07 | 19.2 | 183 | 118 | 1.55 | 1.0793 | 0.0268 | 0.1201 | 0.0011 | 743.3 | 13.0748 | 731.1 | 6.1631 | 98% |
| **BS3** |  |  |  |  |  |  |  |  |  |  |  |  |  |
| BS3-22 | 5.3 | 392 | 214 | 1.83 | 0.1210 | 0.0050 | 0.0165 | 0.0002 | 116.0 | 4.5296 | 105.2 | 1.3739 | 90% |
| BS3-05 | 4.5 | 384 | 167 | 2.30 | 0.1175 | 0.0069 | 0.0167 | 0.0003 | 112.8 | 6.2583 | 106.8 | 1.7677 | 94% |
| BS3-14 | 23.8 | 1011 | 1207 | 0.84 | 0.1136 | 0.0030 | 0.0172 | 0.0002 | 109.2 | 2.7326 | 109.8 | 0.9947 | 99% |
| BS3-13 | 9.5 | 828 | 350 | 2.37 | 0.1099 | 0.0047 | 0.0174 | 0.0002 | 105.9 | 4.3296 | 111.0 | 1.4818 | 95% |
| BS3-25 | 32.4 | 1233 | 1151 | 1.07 | 0.1475 | 0.0033 | 0.0221 | 0.0002 | 139.7 | 2.9326 | 141.0 | 1.2061 | 99% |
| BS3-03 | 19.0 | 948 | 630 | 1.50 | 0.1650 | 0.0055 | 0.0228 | 0.0002 | 155.1 | 4.8343 | 145.3 | 1.2686 | 93% |
| BS3-11 | 71.8 | 4704 | 2021 | 2.33 | 0.1522 | 0.0030 | 0.0229 | 0.0002 | 143.8 | 2.6719 | 145.7 | 1.0114 | 98% |
| BS3-04 | 19.9 | 594 | 739 | 0.80 | 0.1687 | 0.0043 | 0.0230 | 0.0002 | 158.3 | 3.7274 | 146.9 | 1.2242 | 92% |
| BS3-09 | 18.9 | 1001 | 540 | 1.85 | 0.1590 | 0.0050 | 0.0240 | 0.0003 | 149.8 | 4.3642 | 152.8 | 1.5923 | 98% |
| BS3-08 | 6.1 | 229 | 201 | 1.14 | 0.1665 | 0.0069 | 0.0240 | 0.0004 | 156.4 | 6.0223 | 153.2 | 2.2910 | 97% |
| BS3-16 | 26.8 | 227 | 698 | 0.32 | 0.2857 | 0.0071 | 0.0372 | 0.0003 | 255.2 | 5.5738 | 235.6 | 2.0614 | 92% |
| BS3-02 | 21.9 | 290 | 522 | 0.55 | 0.2684 | 0.0064 | 0.0386 | 0.0003 | 241.4 | 5.1189 | 244.4 | 2.1625 | 98% |
| BS3-07 | 45.6 | 164 | 879 | 0.19 | 0.3981 | 0.0081 | 0.0529 | 0.0004 | 340.2 | 5.8523 | 332.2 | 2.5788 | 97% |
| BS3-20 | 18.4 | 220 | 277 | 0.79 | 0.4262 | 0.0099 | 0.0546 | 0.0004 | 360.5 | 7.0623 | 342.9 | 2.5248 | 95% |
| BS3-26 | 72.1 | 416 | 1021 | 0.41 | 0.4953 | 0.0088 | 0.0643 | 0.0004 | 408.5 | 5.9550 | 401.8 | 2.7231 | 98% |
| BS3-24 | 28.7 | 369 | 250 | 1.48 | 0.7129 | 0.0137 | 0.0798 | 0.0006 | 546.4 | 8.1161 | 494.8 | 3.3749 | 90% |
| BS3-21 | 168.7 | 219 | 496 | 0.44 | 4.6057 | 0.0560 | 0.2948 | 0.0018 | 1750.3 | 10.2171 | 1665.5 | 9.1281 | 95% |
| BS3-10 | 83.3 | 126 | 151 | 0.84 | 9.7229 | 0.1495 | 0.4519 | 0.0035 | 2408.9 | 14.2531 | 2403.6 | 15.7465 | 99% |
| BS3-19 | 70.1 | 123 | 110 | 1.11 | 10.9232 | 0.1419 | 0.4690 | 0.0033 | 2516.6 | 12.2021 | 2479.2 | 14.3867 | 98% |
| BS3-01 | 19.3 | 8 | 5 | 1.73 | 135.5698 | 4.9765 | 1.2132 | 0.0475 | 4992.5 | 37.1554 | 5121.3 | 138.4352 | 97% |
| BS3-15* | 27.1 | 487 | 736 | 0.66 | 0.2734 | 0.0075 | 0.0330 | 0.0003 | 245.4 | 5.9505 | 209.1 | 1.8999 | 84% |
| BS3-23* | 5.3 | 79 | 99 | 0.80 | 0.4018 | 0.0150 | 0.0422 | 0.0005 | 342.9 | 10.8828 | 266.5 | 3.3575 | 74% |
| BS3-12* | 12.4 | 89 | 213 | 0.42 | 0.6208 | 0.0344 | 0.0514 | 0.0006 | 490.4 | 21.5472 | 322.9 | 3.5895 | 58% |
| BS3-17* | 30.0 | 359 | 566 | 0.63 | 1.4322 | 0.4326 | 0.0857 | 0.0164 | 902.4 | 180.6060 | 530.3 | 97.3012 | 48% |
| BS3-18* | 9.8 | 804 | 368 | 2.18 | 0.3765 | 0.1165 | 0.0235 | 0.0030 | 324.5 | 85.9568 | 149.8 | 19.2011 | 26% |
| BS3-06* | 3.9 | 187 | 134 | 1.40 | 0.3458 | 0.0282 | 0.0190 | 0.0004 | 301.5 | 21.2760 | 121.1 | 2.7516 | 14% |

*Note:* Zircon analysis results with * were not used in the final age calculation due to too low of their concordance (< 85%)

**Supplementary Table S2.** High-temperature component (HTC) direction of the Meiriqieco Formation volcanic rocks from the Gerze area in the western Qiangtang terrane.

| Sample | Temp (℃) | N | D_g_ (°) | I_g_ (°) | D_s_ (°) | I_s_ (°) | MAD (°) |
| --- | --- | --- | --- | --- | --- | --- | --- |
| **ZL1** |  |  |  |  |  |  |  |
| ZL1-10A | 450-670℃ | 9 | 352.8 | -41.1 | 348.9 | 33.2 | 1.6 |
| ZL1-1A | 400-680℃ | 14 | 354.5 | -36.9 | 353.6 | 35.2 | 1.4 |
| ZL1-2A | 450-680℃ | 14 | 353.6 | -38.8 | 351.3 | 34.4 | 1.4 |
| ZL1-3A | 500-680℃ | 13 | 335.6 | -41.7 | 335.1 | 39.7 | 1.0 |
| ZL1-6A | 400-680℃ | 12 | 352.7 | -43.1 | 347.2 | 31.7 | 1.4 |
| ZL1-7A | 450-680℃ | 11 | 355.8 | -41.4 | 350.6 | 31.3 | 1.9 |
| ZL1-9A | 400-680℃ | 13 | 357.6 | -40.8 | 352.2 | 30.7 | 1.2 |
| **ZL2** |  |  |  |  |  |  |  |
| ZL2-10A | 450-680℃ | 11 | 356.9 | -41.7 | 350.0 | 28.3 | 1.7 |
| ZL2-2A | 560-680℃ | 9 | 352.5 | -42.6 | 346.5 | 29.9 | 2.0 |
| ZL2-3A | 580-680℃ | 7 | 354.5 | -40.2 | 349.7 | 30.7 | 3.0 |
| ZL2-6A | 480-680℃ | 13 | 353.9 | -45.4 | 345.3 | 27.2 | 1.2 |
| ZL2-8A | 500-680℃ | 10 | 359.2 | -44.1 | 349.3 | 25.5 | 1.6 |
| **ZL3** |  |  |  |  |  |  |  |
| ZL3-1A | 450-670℃ | 12 | 348.1 | -38.0 | 348.4 | 38.5 | 1.5 |
| ZL3-2A | 450-670℃ | 12 | 352.4 | -35.6 | 353.5 | 37.4 | 1.4 |
| ZL3-5A | 500-680℃ | 10 | 348.7 | -40.9 | 346.2 | 36.2 | 1.5 |
| ZL3-6A | 500-670℃ | 11 | 356.0 | -42.3 | 349.1 | 31.2 | 2.3 |
| ZL3-7A | 500-670℃ | 8 | 353.9 | -47.6 | 343.3 | 28.9 | 3.0 |
| ZL3-8A | 450-670℃ | 11 | 349.5 | -40.1 | 347.4 | 36.3 | 3.1 |
| ZL3-9A | 450-670℃ | 12 | 350.0 | -37.7 | 350.0 | 37.6 | 1.6 |
| **ZL4** |  |  |  |  |  |  |  |
| ZL4-1A | 500-660℃ | 8 | 355.1 | -43.0 | 342.6 | 24.3 | 2.5 |
| ZL4-2A | 500-680℃ | 9 | 344.9 | -42.8 | 337.9 | 30.4 | 3.2 |
| ZL4-3A | 450-660℃ | 10 | 356.8 | -37.6 | 348.5 | 25.9 | 3.1 |
| ZL4-5A | 480-660℃ | 11 | 350.4 | -39.1 | 344.1 | 29.3 | 2.0 |
| ZL4-6A | 500-680℃ | 12 | 352.9 | -38.9 | 345.5 | 27.8 | 1.3 |
| ZL4-7A | 450-670℃ | 12 | 359.5 | -37.8 | 349.4 | 23.8 | 2.9 |
| ZL4-8A | 500-680℃ | 9 | 353.7 | -37.5 | 347.2 | 28.0 | 2.8 |
| ZL4-9A | 450-660℃ | 12 | 353.5 | -34.9 | 349.7 | 29.4 | 3.7 |
| **ZL5** |  |  |  |  |  |  |  |
| ZL5-10A | 400-600℃ | 8 | 355.5 | -38.4 | 354.5 | 36.6 | 3.1 |
| ZL5-3A | 480-680℃ | 15 | 359.1 | -35.9 | 359.2 | 36.1 | 1.5 |
| ZL5-4A | 540-680℃ | 15 | 357.7 | -40.1 | 354.3 | 34.3 | 1.2 |
| ZL5-6A | 450-680℃ | 17 | 356.3 | -35.7 | 357.7 | 37.9 | 5.6 |
| ZL5-7A | 480-680℃ | 15 | 358.8 | -40.2 | 354.9 | 33.5 | 1.9 |
| ZL5-8A | 550-660℃ | 12 | 353.4 | -36.0 | 355.4 | 39.5 | 2.4 |
| ZL5-9A | 450-680℃ | 13 | 355.1 | -38.8 | 353.9 | 36.6 | 2.9 |
| **ZL6** |  |  |  |  |  |  |  |
| ZL6-1A | 550-640℃ | 10 | 0.1 | -36.6 | 360.0 | 36.4 | 2.3 |
| ZL6-2A | 550-650℃ | 11 | 0.0 | -34.2 | 2.4 | 37.9 | 3.4 |
| ZL6-3A | 560-670℃ | 12 | 1.2 | -31.6 | 5.8 | 38.4 | 4.7 |
| ZL6-4A | 500-660°C | 11 | 355.2 | -36.1 | 357.3 | 39.7 | 1.6 |
| ZL6-6B | 500-620°C | 8 | 1.6 | -33.3 | 4.2 | 37.3 | 4.1 |
| ZL6-8A | 450-620℃ | 9 | 0.1 | -38.7 | 357.9 | 35.1 | 1.6 |
| **ZL7** |  |  |  |  |  |  |  |
| ZL7-10A | 300-540℃ | 6 | 356.1 | -24.3 | 5.3 | 38.4 | 1.3 |
| ZL7-2A | 540-650℃ | 7 | 15.6 | -33.5 | 4.9 | 19.0 | 2.0 |
| ZL7-3A | 520-680℃ | 11 | 8.9 | -28.1 | 7.8 | 26.5 | 1.3 |
| ZL7-4A | 500-680℃ | 12 | 7.9 | -28.7 | 6.8 | 27.0 | 1.5 |
| ZL7-7A | 520-680℃ | 11 | 10.0 | -31.3 | 5.0 | 24.2 | 1.4 |
| ZL7-8A | 480-680℃ | 13 | 357.0 | -32.9 | 357.0 | 32.9 | 0.9 |
| ZL7-9A | 500-680℃ | 10 | 7.7 | -31.1 | 4.2 | 26.2 | 1.2 |
| **ZL8** |  |  |  |  |  |  |  |
| ZL8-10A | 500-680℃ | 12 | 2.8 | -35.1 | 359.4 | 29.8 | 1.2 |
| ZL8-1A | 450-670℃ | 12 | 2.7 | -36.5 | 358.0 | 29.1 | 0.7 |
| ZL8-2A | 450-680℃ | 13 | 15.1 | -40.7 | 359.3 | 18.6 | 0.8 |
| ZL8-3A | 450-680℃ | 14 | 2.5 | -36.1 | 358.3 | 29.4 | 1.1 |
| ZL8-5A | 450-680℃ | 14 | 354.0 | -24.6 | 4.9 | 42.0 | 0.6 |
| ZL8-9A | 500-670℃ | 11 | 358.8 | -37.3 | 355.1 | 31.1 | 0.8 |
| **ZL9** |  |  |  |  |  |  |  |
| ZL9-1A | 500-680℃ | 16 | 2.8 | -35.0 | 5.2 | 38.9 | 1.0 |
| ZL9-2A | 520-680℃ | 14 | 5.8 | -34.3 | 7.8 | 37.3 | 1.5 |
| ZL9-3A | 520-680℃ | 14 | 13.4 | -33.4 | 12.5 | 32.3 | 1.3 |
| ZL9-4A | 500-680℃ | 13 | 3.3 | -41.1 | 359.5 | 34.9 | 1.7 |
| ZL9-5A | 520-680℃ | 14 | 3.2 | -37.9 | 2.6 | 36.9 | 2.1 |
| ZL9-6A | 520-680℃ | 13 | 11.5 | -39.1 | 5.8 | 30.9 | 3.8 |
| ZL9-8A | 550-680℃ | 10 | 1.7 | -37.6 | 2.0 | 38.0 | 1.0 |
| ZL9-9A | 520-680℃ | 13 | 2.9 | -34.9 | 5.5 | 38.9 | 3.0 |
| **ZL10** |  |  |  |  |  |  |  |
| ZL10-11A | 540-670℃ | 9 | 3.9 | -35.2 | 359.7 | 29.6 | 1.2 |
| ZL10-1A | 540-670℃ | 10 | 5.7 | -33.9 | 1.7 | 28.8 | 0.6 |
| ZL10-2A | 450-680℃ | 14 | 3.5 | -32.4 | 2.5 | 31.1 | 2.5 |
| ZL10-3A | 520-670℃ | 11 | 5.6 | -35.1 | 0.4 | 28.4 | 1.7 |
| ZL10-5A | 450-680℃ | 14 | 1.2 | -31.0 | 3.0 | 33.4 | 2.1 |
| ZL10-7A | 500-670℃ | 10 | 6.6 | -35.3 | 0.6 | 27.6 | 1.6 |
| ZL10-8A | 400-680℃ | 12 | 10.6 | -36.7 | 0.5 | 24.0 | 1.4 |
| ZL10-9A | 450-680℃ | 10 | 3.0 | -33.9 | 0.7 | 30.8 | 1.2 |
| **ZL11** |  |  |  |  |  |  |  |
| ZL11-1A | 480-680℃ | 12 | 19.6 | -16.2 | 26.3 | 24.0 | 1.0 |
| ZL11-2A | 400-680℃ | 13 | 21.6 | -14.6 | 28.4 | 22.4 | 1.5 |
| ZL11-5A | 400-680℃ | 13 | 16.2 | -18.0 | 23.6 | 26.8 | 1.3 |
| ZL11-5B | 400-680℃ | 12 | 18.2 | -17.2 | 24.9 | 25.1 | 3.1 |
| ZL11-8A | 480-680℃ | 12 | 27.3 | -13.8 | 30.2 | 17.1 | 1.9 |
| **ZL12** |  |  |  |  |  |  |  |
| ZL12-1A | 400-680℃ | 14 | 21.5 | -14.6 | 28.4 | 22.5 | 1.2 |
| ZL12-4A | 500-680℃ | 12 | 16.8 | -14.0 | 28.0 | 27.1 | 1.3 |
| ZL12-6A | 450-680℃ | 13 | 17.2 | -11.3 | 31.1 | 27.3 | 0.8 |
| ZL12-7A | 450-680℃ | 13 | 14.3 | -17.8 | 23.3 | 28.6 | 1.3 |
| ZL12-8A | 450-680℃ | 11 | 21.1 | -15.1 | 27.7 | 22.8 | 2.3 |
| **ZL13** |  |  |  |  |  |  |  |
| ZL13-10A | 400-680℃ | 11 | 346.1 | -12.9 | 16.0 | 55.6 | 0.8 |
| ZL13-11A | 500-680℃ | 10 | 359.9 | -10.3 | 27.5 | 44.0 | 2.1 |
| ZL13-1A | 400-680℃ | 14 | 18.0 | -13.3 | 29.1 | 26.0 | 1.3 |
| ZL13-2A | 520-680℃ | 9 | 11.5 | -7.9 | 33.8 | 33.4 | 1.7 |
| ZL13-4A | 450-680℃ | 14 | 16.4 | -13.8 | 28.2 | 27.5 | 0.9 |
| ZL13-5A | 400-680℃ | 14 | 9.9 | -4.2 | 37.9 | 35.5 | 1.0 |
| ZL13-6A | 450-680℃ | 14 | 9.0 | -14.4 | 25.5 | 34.4 | 1.3 |
| ZL13-7A | 400-680℃ | 12 | 17.3 | -18.9 | 22.8 | 25.5 | 1.3 |
| **ZL14** |  |  |  |  |  |  |  |
| ZL14-2A | 520-680℃ | 16 | 2.7 | -42.0 | 353.6 | 27.5 | 3.5 |
| ZL14-3A | 480-680℃ | 15 | 359.9 | -43.7 | 350.6 | 28.1 | 3.4 |
| ZL14-4A | 520-670℃ | 13 | 3.1 | -39.9 | 355.8 | 28.5 | 2.2 |
| ZL14-5A | 500-680℃ | 16 | 1.4 | -39.1 | 355.6 | 29.9 | 2.2 |
| ZL14-6A | 520-600℃ | 8 | 4.8 | -40.3 | 356.1 | 27.1 | 2.5 |
| ZL14-7A | 500-680℃ | 12 | 359.4 | -42.4 | 351.5 | 29.2 | 2.2 |
| ZL14-8A | 540-680℃ | 11 | 0.4 | -39.7 | 354.5 | 30.2 | 4.9 |
| **ZL15** |  |  |  |  |  |  |  |
| ZL15-2A | 620-670℃ | 5 | 12.2 | -53.5 | 346.9 | 15.9 | 7.0 |
| ZL15-3A | 650-680℃ | 4 | 3.6 | -47.2 | 349.3 | 23.9 | 6.9 |
| ZL15-4A | 640-680℃ | 5 | 353.8 | -51.6 | 341.0 | 25.8 | 6.6 |
| ZL15-7A | 620-680℃ | 5 | 359.1 | -28.1 | 5.5 | 37.6 | 3.9 |
| ZL15-8A | 620-680℃ | 5 | 353.5 | -29.7 | 0.2 | 40.7 | 6.1 |
| ZL15-9A | 560-600℃ | 5 | 347.1 | -27.3 | 358.0 | 46.7 | 7.9 |
| **ZL16** |  |  |  |  |  |  |  |
| ZL16-2A | 500-680℃ | 12 | 353.4 | -33.3 | 356.5 | 38.6 | 2.3 |
| ZL16-3A | 500-680℃ | 12 | 351.6 | -35.7 | 352.9 | 38.2 | 2.8 |
| ZL16-4A | 450-660℃ | 12 | 350.5 | -33.4 | 354.4 | 40.4 | 1.8 |
| ZL16-5A | 500-680℃ | 12 | 356.1 | -32.5 | 359.1 | 37.3 | 2.8 |
| ZL16-6A | 540-680℃ | 9 | 352.7 | -31.5 | 357.8 | 40.2 | 2.2 |
| ZL16-7A | 500-680℃ | 10 | 352.6 | -36.5 | 352.9 | 37.0 | 2.7 |
| ZL16-8A | 500-600℃ | 9 | 348.8 | -37.0 | 349.8 | 38.9 | 3.6 |
| ZL16-9A | 500-680℃ | 10 | 353.8 | -35.2 | 355.0 | 37.2 | 1.5 |
| **ZL17** |  |  |  |  |  |  |  |
| ZL17-1A | 450-670℃ | 12 | 353.3 | -36.5 | 353.4 | 36.6 | 1.8 |
| ZL17-2A | 450-660℃ | 12 | 349.1 | -34.2 | 352.6 | 40.7 | 2.5 |
| ZL17-3A | 450-680℃ | 13 | 346.8 | -36.5 | 348.7 | 40.3 | 2.2 |
| ZL17-6A | 450-650℃ | 10 | 352.0 | -33.7 | 355.1 | 39.3 | 2.7 |
| ZL17-7A | 500-680℃ | 8 | 351.4 | -30.9 | 357.6 | 41.4 | 2.8 |
| ZL17-8A | 450-660℃ | 10 | 349.1 | -30.0 | 356.7 | 43.6 | 3.2 |
| ZL17-9A | 520-680℃ | 10 | 356.7 | -35.9 | 356.1 | 34.9 | 2.1 |
| **ZL18** |  |  |  |  |  |  |  |
| ZL18-10A | 500-680℃ | 9 | 355.0 | -31.7 | 359.2 | 38.5 | 2.5 |
| ZL18-1A | 450-670℃ | 12 | 353.4 | -35.7 | 354.2 | 37.0 | 3.6 |
| ZL18-2A | 450-650℃ | 10 | 348.7 | -33.3 | 353.2 | 41.6 | 3.5 |
| ZL18-3A | 480-680℃ | 11 | 353.0 | -37.5 | 352.2 | 36.1 | 1.2 |
| ZL18-5A | 480-680℃ | 13 | 357.8 | -34.0 | 358.6 | 35.2 | 2.0 |
| ZL18-6A | 350-650℃ | 13 | 356.1 | -36.5 | 355.2 | 34.9 | 3.3 |
| ZL18-7A | 400-640℃ | 11 | 353.4 | -37.7 | 352.3 | 35.8 | 2.3 |
| **ZL19** |  |  |  |  |  |  |  |
| ZL19-2A | 500-660℃ | 10 | 353.5 | -32.3 | 357.5 | 39.1 | 3.1 |
| ZL19-3A | 500-670℃ | 11 | 352.7 | -32.0 | 357.3 | 39.9 | 3.2 |
| ZL19-4A | 450-680℃ | 13 | 350.7 | -32.4 | 355.5 | 40.9 | 1.4 |
| ZL19-5A | 400-640℃ | 11 | 354.0 | -32.3 | 358.0 | 38.9 | 2.5 |
| ZL19-6A | 500-660℃ | 10 | 352.3 | -33.3 | 355.7 | 39.3 | 3.1 |
| ZL19-7A | 480-680℃ | 13 | 348.2 | -35.6 | 350.6 | 40.2 | 2.4 |
| ZL19-9A | 450-670℃ | 8 | 348.6 | -35.9 | 350.6 | 39.8 | 1.7 |
| **ZL20** |  |  |  |  |  |  |  |
| ZL20-10A | 480-660℃ | 11 | 352.0 | -31.4 | 357.4 | 40.7 | 2.3 |
| ZL20-11A | 450-660℃ | 12 | 3.3 | -33.1 | 2.5 | 32.0 | 1.4 |
| ZL20-2A | 450-660℃ | 12 | 357.5 | -36.0 | 356.5 | 34.3 | 1.8 |
| ZL20-3A | 450-660℃ | 12 | 354.7 | -36.6 | 354.1 | 35.7 | 1.3 |
| ZL20-6A | 500-670℃ | 9 | 9.5 | -39.5 | 358.9 | 24.4 | 2.6 |
| ZL20-8A | 450-580℃ | 8 | 357.2 | -14.7 | 20.5 | 45.1 | 4.6 |
| ZL20-9A | 450-660℃ | 8 | 352.7 | -21.5 | 9.2 | 45.9 | 1.8 |
| **ZL21** |  |  |  |  |  |  |  |
| ZL21-10A | 450-670℃ | 13 | 353.0 | -32.3 | 357.2 | 39.5 | 1.3 |
| ZL21-12A | 480-660℃ | 11 | 358.8 | -31.8 | 1.4 | 35.8 | 2.1 |
| ZL21-13A | 540-680℃ | 10 | 356.8 | -33.9 | 358.0 | 36.0 | 1.5 |
| ZL21-1A | 480-660℃ | 11 | 346.4 | -28.8 | 355.7 | 46.0 | 2.7 |
| ZL21-2A | 560-660℃ | 8 | 349.5 | -31.9 | 355.1 | 42.0 | 1.3 |
| ZL21-3A | 480-660℃ | 11 | 347.3 | -38.2 | 347.6 | 38.9 | 1.8 |
| ZL21-5A | 450-660℃ | 12 | 350.0 | -37.7 | 350.0 | 37.7 | 1.8 |
| ZL21-9A | 480-660℃ | 11 | 353.6 | -33.1 | 356.9 | 38.6 | 2.8 |
| **ZL22** |  |  |  |  |  |  |  |
| ZL22-10A | 560-680℃ | 8 | 139.5 | -20.4 | 304.4 | -73.3 | 3.9 |
| ZL22-1A | 450-670℃ | 13 | 135.2 | -5.0 | 355.3 | -88.7 | 1.1 |
| ZL22-2A | 450-680℃ | 14 | 139.5 | -8.4 | 278.0 | -84.4 | 1.1 |
| ZL22-3A | 400-680℃ | 12 | 134.3 | -4.5 | 28.7 | -88.2 | 1.1 |
| ZL22-4A | 450-680℃ | 11 | 190.0 | -23.6 | 252.0 | -34.4 | 0.7 |
| ZL22-5A | 400-680℃ | 12 | 149.6 | -14.9 | 266.0 | -72.7 | 0.9 |
| ZL22-6A | 400-680℃ | 12 | 178.8 | -14.1 | 242.4 | -46.6 | 2.0 |
| ZL22-7A | 500-670℃ | 12 | 147.0 | -15.2 | 272.7 | -74.4 | 0.7 |
| ZL22-9A | 520-580℃ | 6 | 180.3 | -11.9 | 238.9 | -45.5 | 1.1 |
| **ZL23** |  |  |  |  |  |  |  |
| ZL23-1A | 480-660℃ | 10 | 14.8 | -37.6 | 9.6 | 29.0 | 4.1 |
| ZL23-3A | 400-670℃ | 15 | 13.6 | -34.0 | 12.4 | 31.9 | 1.7 |
| ZL23-4A | 400-670℃ | 13 | 14.6 | -35.0 | 11.9 | 30.6 | 2.3 |
| ZL23-5A | 450-680℃ | 14 | 18.3 | -38.9 | 10.3 | 26.0 | 1.3 |
| ZL23-6A | 450-670℃ | 13 | 9.3 | -39.3 | 4.9 | 31.1 | 0.9 |
| ZL23-7A | 450-680℃ | 13 | 12.0 | -35.7 | 9.8 | 31.9 | 0.8 |
| ZL23-9A | 450-680℃ | 12 | 17.3 | -36.3 | 12.1 | 28.0 | 0.8 |
| **ZL24** |  |  |  |  |  |  |  |
| ZL24-10A | 450-680℃ | 9 | 16.0 | -35.7 | 12.0 | 29.3 | 2.7 |
| ZL24-1A | 400-660℃ | 13 | 16.8 | -37.2 | 11.0 | 27.9 | 1.9 |
| ZL24-3A | 450-660℃ | 12 | 19.0 | -37.2 | 12.2 | 26.4 | 1.5 |
| ZL24-7A | 450-680℃ | 11 | 21.6 | -36.3 | 14.2 | 25.1 | 3.0 |
| ZL24-8A | 520-650℃ | 9 | 16.8 | -33.7 | 14.4 | 29.8 | 1.7 |
| ZL24-9A | 480-660℃ | 11 | 24.4 | -36.2 | 15.5 | 23.2 | 3.8 |
| **ZL25** |  |  |  |  |  |  |  |
| ZL25-3A | 400-680℃ | 12 | 23.8 | -37.1 | 14.5 | 23.2 | 1.0 |
| ZL25-5A | 400-680℃ | 14 | 8.9 | -36.4 | 7.2 | 33.3 | 0.6 |
| ZL25-6A | 400-680℃ | 16 | 23.7 | -34.6 | 16.8 | 24.5 | 1.0 |
| ZL25-7A | 400-680℃ | 12 | 5.9 | -36.2 | 5.4 | 35.2 | 0.9 |
| ZL25-8A | 400-680℃ | 15 | 17.8 | -37.4 | 11.4 | 27.1 | 0.6 |
| ZL25-9A | 350-680℃ | 16 | 14.3 | -37.0 | 9.9 | 29.7 | 0.9 |
| **ZL26** |  |  |  |  |  |  |  |
| ZL26-2A | 350-680℃ | 17 | 22.1 | -35.7 | 15.0 | 25.0 | 1.4 |
| ZL26-3A | 450-670℃ | 13 | 10.6 | -34.3 | 10.2 | 33.7 | 1.0 |
| ZL26-4A | 450-680℃ | 14 | 15.2 | -36.6 | 10.8 | 29.2 | 0.8 |
| ZL26-5A | 450-670℃ | 12 | 22.4 | -36.3 | 14.6 | 24.6 | 1.9 |
| ZL26-7A | 500-650℃ | 10 | 13.8 | -33.4 | 12.9 | 32.1 | 2.1 |
| ZL26-9A | 450-670℃ | 9 | 347.9 | -26.8 | 356.5 | 52.0 | 3.2 |
| **ZL27** |  |  |  |  |  |  |  |
| ZL27-1A | 520-650℃ | 9 | 15.0 | -38.1 | 9.3 | 28.5 | 2.8 |
| ZL27-5A | 520-660℃ | 9 | 15.9 | -32.2 | 15.4 | 31.3 | 2.8 |
| ZL27-6A | 540-680℃ | 10 | 16.4 | -41.9 | 6.6 | 25.4 | 3.5 |
| ZL27-8A | 450-680℃ | 11 | 13.7 | -33.1 | 13.2 | 32.3 | 2.4 |
| ZL27-9A | 540-680℃ | 9 | 19.1 | -35.1 | 14.2 | 27.5 | 2.6 |
| **ZL28** |  |  |  |  |  |  |  |
| ZL28-10A | 450-680℃ | 11 | 20.8 | -36.8 | 13.4 | 25.4 | 1.0 |
| ZL28-11A | 450-680℃ | 14 | 11.6 | -37.5 | 7.9 | 31.0 | 0.8 |
| ZL28-4A | 450-680℃ | 11 | 7.9 | -35.2 | 7.6 | 34.7 | 1.4 |
| ZL28-5A | 450-650℃ | 11 | 41.8 | -35.5 | 21.9 | 10.4 | 2.6 |
| ZL28-6A | 450-670℃ | 13 | 21.8 | -31.0 | 19.4 | 27.6 | 2.0 |
| **ZL29** |  |  |  |  |  |  |  |
| ZL29-10A | 480-660℃ | 11 | 16.5 | -38.3 | 9.9 | 27.5 | 2.3 |
| ZL29-1A | 450-660℃ | 12 | 21.1 | -36.3 | 13.9 | 25.5 | 2.6 |
| ZL29-2A | 450-660℃ | 12 | 16.0 | -38.3 | 9.7 | 27.7 | 2.2 |
| ZL29-7A | 400-660℃ | 13 | 12.0 | -37.6 | 8.0 | 30.7 | 0.6 |
| ZL29-8A | 400-650℃ | 12 | 17.9 | -35.8 | 12.9 | 28.0 | 1.2 |
| ZL29-9A | 400-680℃ | 12 | 19.5 | -34.6 | 14.9 | 27.5 | 0.9 |
| **ZL30** |  |  |  |  |  |  |  |
| ZL30-10A | 450-680℃ | 11 | 46.4 | -33.4 | 24.9 | 7.3 | 1.0 |
| ZL30-12A | 540-680℃ | 9 | 24.3 | -36.4 | 15.3 | 23.2 | 2.0 |
| ZL30-2A | 500-680℃ | 12 | 17.7 | -37.6 | 11.2 | 27.0 | 2.7 |
| ZL30-5A | 450-650℃ | 11 | 17.4 | -33.0 | 15.3 | 29.8 | 2.0 |
| ZL30-8A | 450-660℃ | 9 | 24.3 | -39.9 | 12.0 | 21.5 | 2.3 |
| **ZL31** |  |  |  |  |  |  |  |
| ZL31-2A | 400-680℃ | 12 | 14.1 | -37.6 | 9.2 | 29.4 | 0.8 |
| ZL31-3A | 400-680℃ | 15 | 14.7 | -36.5 | 10.6 | 29.7 | 0.7 |
| ZL31-4A | 480-660℃ | 11 | 21.0 | -38.3 | 12.1 | 24.5 | 0.7 |
| ZL31-5A | 450-680℃ | 11 | 16.1 | -34.9 | 12.9 | 29.7 | 0.5 |
| ZL31-6A | 450-650℃ | 11 | 17.7 | -37.5 | 11.3 | 27.1 | 0.8 |
| ZL31-7A | 450-650℃ | 11 | 2.1 | -28.5 | 9.6 | 43.0 | 1.1 |
| ZL31-8A | 450-680℃ | 11 | 15.9 | -36.4 | 11.3 | 28.9 | 0.8 |

*Notes:* Temp, temperature; N, number of consecutive demagnetization steps used to define the HTC direction; D_g_ and I_g_, declination and inclination in geographic coordinates; D_s_ and I_s_, declination and inclination in stratigraphic coordinates; MAD, mean angular deviation of the HTC fit.

**Supplementary Table S3.** Site(Group)-mean HTC directions of the Meiriqieco Formation volcanic rocks from the Gerze area in the western Qiangtang terrane.

| Direction group | Site ID | Strike/Dip | n/N | D_g_ | I_g_ | D_s_ | I_s_ | k | α_95_ | Plat | Plon |
| --- | --- | --- | --- | --- | --- | --- | --- | --- | --- | --- | --- |
|  |  | (°) |  | (°) | (°) | (°) | (°) |  | (°) | (°) | (°) |
| This study | | | | | | | | | | | |
| D1 | ZL1 | 48/84 | 7/9 | 351.9 | -40.7 | 348.6 | 33.9 | 188.7 | 4.4 | 72.6 | 302.1 |
| D2 | ZL2 | 47/82 | 5/8 | 355.4 | -42.8 | 348.2 | 28.3 | 835.2 | 2.6 | 69.4 | 297.5 |
| D3 | ZL3 | 43/88 | 7/7 | 351.2 | -40.3 | 348.2 | 35.2 | 324.8 | 3.4 | 73.5 | 301.8 |
| D4 | ZL4 | 34/86 | 8/9 | 353.4 | -39.0 | 345.6 | 27.4 | 350.2 | 3.0 | 67.6 | 302.6 |
| D5 | ZL5 | 49/87 | 7/8 | 356.5 | -37.9 | 355.7 | 36.4 | 985.2 | 1.9 | 77.0 | 281.6 |
| D6 | ZL6 | 50/88 | 6/7 | 359.7 | -35.1 | 1.3 | 37.5 | 654.2 | 2.6 | 78.2 | 257.4 |
| D7 | ZL7 | 44/83 | 7/8 | 6.1 | -30.1 | 4.5 | 27.8 | 137.5 | 5.2 | 71.6 | 249.5 |
| D8 | ZL8 | 46/84 | 6/7 | 2.3 | -35.2 | 359.0 | 30.0 | 106.0 | 6.5 | 73.4 | 266.8 |
| D9 | ZL9 | 54/89 | 8/8 | 5.6 | -36.7 | 5.2 | 36.1 | 330.9 | 3.0 | 76.5 | 242.0 |
| D10 | ZL10 | 42/89 | 8/8 | 5.0 | -34.2 | 1.1 | 29.2 | 748.9 | 2.0 | 72.9 | 259.8 |
| D11 | ZL11 | 46/86 | 5/6 | 20.6 | -16.0 | 26.7 | 23.1 | 332.6 | 4.2 | 58.0 | 207.4 |
| D12 | ZL12 | 46/86 | 5/6 | 18.2 | -14.6 | 27.7 | 25.7 | 464.2 | 3.6 | 58.3 | 204.1 |
| D13 | ZL13 | 46/86 | 8/9 | 8.5 | -12.1 | 28.0 | 35.3 | 50.5 | 7.9 | 61.7 | 194.3 |
| D14 | ZL14 | 46/86 | 7/8 | 1.7 | -41.0 | 354.0 | 28.7 | 999.9 | 1.7 | 71.8 | 282.2 |
| D15 | ZL15 | 46/86 | 6/8 | 357.3 | -39.8 | 352.7 | 32.1 | 33.4 | 11.8 | 73.4 | 288.4 |
|  | ZL16 | 46/86 | 8/8 | 352.5 | -34.4 | 354.8 | 38.5 | 905.1 | 1.8 | 78.1 | 287.4 |
|  | ZL17 | 46/86 | 7/7 | 351.2 | -34.0 | 354.3 | 39.6 | 465.5 | 2.8 | 78.6 | 291.1 |
|  | ZL18 | 46/86 | 7/7 | 353.9 | -35.2 | 355.0 | 37.0 | 611.7 | 2.4 | 77.1 | 284.9 |
|  | ZL19 | 46/86 | 7/8 | 351.5 | -33.4 | 355.0 | 39.8 | 999.9 | 1.9 | 79.0 | 288.3 |
| D16 | ZL16+17+18+19 | 46/86 | 29/30 | 352.3 | -34.3 | 354.8 | 38.7 | 674.7 | 1.0 | 78.2 | 287.7 |
| D17 | ZL20 | 46/86 | 7/8 | 357.9 | -30.5 | 2.2 | 37.2 | 60.6 | 7.8 | 77.9 | 253.5 |
| D18 | ZL21 | 46/86 | 8/8 | 351.9 | -33.5 | 355.3 | 39.4 | 279.3 | 3.3 | 78.8 | 286.4 |
|  | *ZL22 | 46/86 | 9/9 | 154.3 | -14.0 | 256.0 | -69.4 | 13.7 | 14.4 | -33.7 | 307.9 |
| D19 | ZL23 | 60/83 | 7/8 | 14.3 | -36.7 | 10.2 | 29.8 | 667.4 | 2.3 | 70.9 | 232.1 |
| D20 | ZL24 | 60/83 | 6/9 | 19.1 | -36.1 | 13.2 | 27.0 | 745.7 | 2.5 | 68.0 | 227.2 |
|  | ZL25 | 60/83 | 6/7 | 15.7 | -36.6 | 11.0 | 28.9 | 177.1 | 5.0 | 70.1 | 230.8 |
|  | ZL26 | 60/83 | 6/7 | 11.7 | -34.4 | 10.8 | 32.8 | 51.3 | 9.4 | 72.3 | 227.6 |
|  | ZL31 | 60/83 | 7/7 | 14.3 | -35.8 | 11.0 | 30.3 | 185.1 | 4.4 | 70.8 | 229.5 |
| D21 | ZL25+26+31 | 60/83 | 19/21 | 13.9 | -35.6 | 11.0 | 30.7 | 108.7 | 3.2 | 71.0 | 229.2 |
|  | ZL27 | 60/83 | 5/8 | 16.0 | -36.1 | 11.7 | 29.0 | 357.4 | 4.1 | 69.8 | 229.0 |
|  | ZL28 | 60/83 | 5/9 | 20.8 | -35.8 | 14.3 | 26.0 | 54.7 | 10.4 | 67.0 | 225.6 |
|  | ZL29 | 60/83 | 6/8 | 17.2 | -36.9 | 11.6 | 27.8 | 760.2 | 2.4 | 69.2 | 230.2 |
| D22 | ZL27+28  +29 | 60/83 | 16/25 | 17.9 | -36.3 | 12.5 | 27.6 | 153.8 | 3.0 | 68.7 | 228.3 |
| D23 | ZL30 | 60/83 | 5/7 | 26.1 | -36.5 | 15.9 | 21.9 | 64.3 | 9.6 | 64.1 | 225.4 |
| Sub-mean | Overall mean A  N = 31 sites | |  | 4.0 | -33.3 | 4.8 | 33.7 | 31.0 | 4.7 | 75.6 | 244.0 |
|  |  |  |  |  |  |  |  |  |  | K=25.5 | A_95_=5.2 |
| Sub-mean | Overall mean B  N = 30 sites | |  | 5.2 | -34.6 | 4.0 | 32.3 | 50.9 | 3.7 | 74.5 | 249.5 |
|  |  |  |  |  |  |  |  |  |  | K=47.5 | A_95_=3.9 |
| Group mean | Overall mean C  N = 23 sites | |  | 5.0 | -34.2 | 3.7 | 31.9 | 45.2 | 4.6 | 74.3 | 250.5 |
|  |  |  |  |  |  |  |  |  |  | K=41.6 | A_95_=4.7 |
| Previous study by Cao et al. (2020) | | | | | | | | | | | |
|  | WK1 | 228/30 | 6/9 | 74.4 | 35.1 | 50.2 | 42.8 | 441.5 | 3.2 | 45.7 | 170.4 |
|  | WK2 | 228/30 | 8/10 | 83.7 | 44.4 | 49.1 | 54.5 | 55.7 | 7.5 | 49.6 | 156.4 |
|  | WK3 | 228/30 | 7/8 | 94.9 | 38.5 | 67.0 | 55.6 | 79.4 | 6.8 | 35.8 | 150.0 |
|  | WK4 | 228/30 | 7/8 | 101.4 | 36.0 | 77.1 | 56.5 | 224.8 | 4.0 | 28.5 | 145.7 |
|  | WK5 | 228/30 | 7/8 | 75.8 | 39.2 | 47.8 | 46.8 | 234.5 | 3.9 | 48.9 | 167.2 |
|  | WK6 | 228/30 | 5/8 | 83.7 | 25.0 | 66.5 | 39.1 | 35.6 | 13.0 | 31.0 | 165.8 |
|  | WK8 | 228/30 | 10/10 | 87.8 | 27.6 | 68.9 | 43.2 | 141.1 | 4.1 | 30.3 | 161.5 |
|  | WK9 | 228/30 | 10/10 | 89.0 | 23.7 | 72.8 | 40.4 | 111.3 | 4.6 | 26.2 | 161.9 |
|  | WK10 | 228/30 | 8/11 | 85.4 | 29.0 | 65.4 | 43.3 | 270.5 | 3.4 | 33.2 | 162.9 |
|  | WK11 | 228/30 | 8/9 | 83.6 | 29.3 | 63.4 | 42.6 | 266.1 | 3.4 | 34.6 | 164.4 |
|  | WK12 | 228/30 | 11/11 | 91.0 | 31.0 | 69.6 | 47.6 | 49.5 | 6.6 | 31.2 | 157.4 |
|  | WK13 | 228/30 | 6/8 | 84.0 | 26.4 | 65.9 | 40.4 | 154.4 | 5.4 | 31.9 | 165.1 |
|  | WK14 | 228/30 | 8/8 | 87.8 | 23.1 | 71.9 | 39.3 | 190.1 | 4.0 | 26.6 | 163.1 |
|  | WK15 | 204/33 | 9/9 | 69.5 | 25.2 | 50.0 | 45.1 | 186.3 | 3.8 | 46.6 | 168.1 |
|  | WK16 | 204/33 | 8/9 | 67.7 | 29.1 | 44.9 | 47.4 | 1361.0 | 1.5 | 51.4 | 167.7 |
|  | WK17 | 209/33 | 9/9 | 64.4 | 27.5 | 42.9 | 41.8 | 233.4 | 3.4 | 51.6 | 175.4 |
| Group mean | Overall mean D  N = 39 sites | |  | 36.5 | -10.2 | 23.9 | 41.0 | 10.6 | 7.4 | 65.9 | 188.0 |
|  |  |  |  |  |  |  |  |  |  | K=8.0 | A_95_=8.6 |
| Previous study by Cao et al. (2020) after a 57.2° counterclockwise rotation | | | | | | | | | | | |
|  | WK1 | 228/30 | 6/9 | 17.2 | 35.1 | 353 | 42.8 | 441.5 | 3.2 | 79.9 | 302.5 |
|  | WK2 | 228/30 | 8/10 | 26.5 | 44.4 | 351.9 | 54.5 | 55.7 | 7.5 | 83.0 | 13.3 |
|  | WK3 | 228/30 | 7/8 | 37.7 | 38.5 | 9.8 | 55.6 | 79.4 | 6.8 | 81.3 | 148.9 |
|  | WK4 | 228/30 | 7/8 | 44.2 | 36.0 | 19.9 | 56.5 | 224.8 | 4.0 | 73.2 | 153.6 |
|  | WK5 | 228/30 | 7/8 | 18.6 | 39.2 | 350.6 | 46.8 | 234.5 | 3.9 | 80.6 | 325.0 |
|  | WK6 | 228/30 | 5/8 | 26.5 | 25.0 | 9.3 | 39.1 | 35.6 | 13.0 | 76.4 | 223.8 |
|  | WK8 | 228/30 | 10/10 | 30.6 | 27.6 | 11.7 | 43.2 | 141.1 | 4.1 | 77.2 | 207.7 |
|  | WK9 | 228/30 | 10/10 | 31.8 | 23.7 | 15.6 | 40.4 | 111.3 | 4.6 | 73.1 | 205.2 |
|  | WK10 | 228/30 | 8/11 | 28.2 | 29.0 | 8.2 | 43.3 | 270.5 | 3.4 | 79.5 | 218.4 |
|  | WK11 | 228/30 | 8/9 | 26.4 | 29.3 | 6.2 | 42.6 | 266.1 | 3.4 | 80.2 | 228.5 |
|  | WK12 | 228/30 | 11/11 | 33.8 | 31.0 | 12.4 | 47.6 | 49.5 | 6.6 | 78.6 | 191.7 |
|  | WK13 | 228/30 | 6/8 | 26.8 | 26.4 | 8.7 | 40.4 | 154.4 | 5.4 | 77.5 | 223.4 |
|  | WK14 | 228/30 | 8/8 | 30.6 | 23.1 | 14.7 | 39.3 | 190.1 | 4.0 | 73.2 | 209.1 |
|  | WK15 | 204/33 | 9/9 | 12.3 | 25.2 | 352.8 | 45.1 | 186.3 | 3.8 | 81.2 | 310.3 |
|  | WK16 | 204/33 | 8/9 | 10.5 | 29.1 | 347.7 | 47.4 | 1361.0 | 1.5 | 78.6 | 334.3 |
|  | WK17 | 209/33 | 9/9 | 7.2 | 27.5 | 345.7 | 41.8 | 233.4 | 3.4 | 74.7 | 322.1 |
| Group mean | Overall mean E  N = 39 sites | |  | 13.6 | -8.2 | 3.7 | 37.7 | 38.9 | 3.7 | 78.3 | 246.5 |
|  |  |  |  |  |  |  |  |  |  | K=40.0 | A_95_=3.7 |

*Notes:* Site ID, site identification; Strike/dip, right hand strike/dip of the beds; n/N, number of samples used to calculate mean and measured; D_g_, I_g_, D_s_, and I_s_, declination and inclination in geographic and stratigraphic coordinates, respectively; k (K), the best estimate of the precision parameter; α_95_ (A_95_), the radius that the mean direction (pole) lies within the 95% confidence; Plat and Plon, latitude and longitude of palaeopoles in stratigraphic coordinates; *ZL22 are identified as an outlier by the Jackknife test^55^. (1) Overall mean D (N = 39 sites without ZL22): The McElhinny^70^ fold test is positive at 95% and 99% confidence levels: k_s_/k_g_ = 3.73 > F(2*(n2-1), (n1-1)) at 5% and 1% point = 1.46 and 1.71, respectively. The McFadden^54^ fold test is positive at 95% and 99% confidence levels at “Xi2” test: critical “Xi” at 95% and 99% = 7.26 and 10.27, respectively. “Xi2” IS = 32.94, “Xi2” TC = 0.17. (2) Overall mean E (N = 39 sites without ZL22): The McElhinny^70^ fold test is positive at 95% and 99% confidence levels: k_s_/k_g_ = 7.23 > F(2*(n2-1), (n1-1)) at 5% and 1% point = 1.46 and 1.71, respectively.

**Supplementary Table S4.** Summary of the Early Cretaceous palaeomagnetic results from the western Lhasa and Qiangtang terranes.

| Site | Plat(°N) | Plon(°E) | Rlat(°N) | Rlon(°E) | Dr(°) | Ir(°) |
| --- | --- | --- | --- | --- | --- | --- |
| The western Qiangtang terrane | | | | | | |
| The Early Cretaceous (~113–109 Ma) volcanic results from the Meiriqieco Formation in the Gerze area of this study. | | | | | | |
| D1 | 72.6 | 302.1 | 32.7 | 83.4 | 348.6 | 34.0 |
| D2 | 69.4 | 297.5 | 32.7 | 83.4 | 348.2 | 28.4 |
| D3 | 73.5 | 301.8 | 32.7 | 83.4 | 349.2 | 35.0 |
| D4 | 67.6 | 302.6 | 32.7 | 83.4 | 345.6 | 27.4 |
| D5 | 77.0 | 281.6 | 32.7 | 83.4 | 355.7 | 36.5 |
| D6 | 78.2 | 257.4 | 32.7 | 83.4 | 1.3 | 37.5 |
| D7 | 71.6 | 249.5 | 32.7 | 83.4 | 4.5 | 27.8 |
| D8 | 73.4 | 266.8 | 32.7 | 83.4 | 359.0 | 30.0 |
| D9 | 76.5 | 242.0 | 32.7 | 83.4 | 5.2 | 36.1 |
| D10 | 72.9 | 259.8 | 32.7 | 83.4 | 1.1 | 29.2 |
| D11 | 58.0 | 207.4 | 32.7 | 83.4 | 26.7 | 23.1 |
| D12 | 58.3 | 204.1 | 32.7 | 83.4 | 27.7 | 25.7 |
| D13 | 61.7 | 194.3 | 32.7 | 83.4 | 28.0 | 35.3 |
| D14 | 71.8 | 282.2 | 32.7 | 83.4 | 354.0 | 28.7 |
| D15 | 73.4 | 288.4 | 32.7 | 83.4 | 352.7 | 32.2 |
| D16 | 78.2 | 287.7 | 32.7 | 83.4 | 354.8 | 38.7 |
| D17 | 77.9 | 253.5 | 32.7 | 83.4 | 2.2 | 37.2 |
| D18 | 78.8 | 286.4 | 32.7 | 83.4 | 355.3 | 39.4 |
| D20 | 70.9 | 232.1 | 32.7 | 83.4 | 10.2 | 29.8 |
| D21 | 68.0 | 227.2 | 32.7 | 83.4 | 13.2 | 27.0 |
| D22 | 71.0 | 229.2 | 32.7 | 83.4 | 11.0 | 30.7 |
| D23 | 68.7 | 228.3 | 32.7 | 83.4 | 12.5 | 27.6 |
| D24 | 64.1 | 225.4 | 32.7 | 83.4 | 15.9 | 21.8 |
| The Early Cretaceous (Albian–Aptian) red sandstone results in the Longmucuo area reported by Chen et al.^40^ | | | | | | |
| 3 | 65.7 | 212.8 | 32.7 | 83.4 | 19.3 | 29.5 |
| 4 | 59.8 | 234.4 | 32.7 | 83.4 | 14.2 | 11.0 |
| 8 | 59.5 | 228.9 | 32.7 | 83.4 | 16.8 | 12.9 |
| 9 | 70.9 | 253.6 | 32.7 | 83.4 | 3.3 | 26.2 |
| The Early Cretaceous (Albian–Aptian) red sandstone results in the Aksaichin area reported by Chen et al.^40^ | | | | | | |
| 41 | 66.5 | 273.6 | 32.7 | 83.4 | 355.9 | 18.5 |
| 42 | 64.6 | 264.5 | 32.7 | 83.4 | 359.5 | 14.4 |
| 44 | 65.4 | 267.3 | 32.7 | 83.4 | 358.4 | 16.0 |
| 45 | 52.6 | 232.5 | 32.7 | 83.4 | 18.2 | -1.1 |
| 46 | 66.2 | 258.0 | 32.7 | 83.4 | 2.2 | 17.6 |
| 48 | 70.5 | 268.7 | 32.7 | 83.4 | 358.2 | 25.3 |
| 49 | 66.4 | 247.1 | 32.7 | 83.4 | 6.5 | 19.2 |
| The Early Cretaceous (~120–115 Ma) volcanic results from the Meiriqieco Formation in the Gerze area reported by Cao et al.^42^ | | | | | | |
| WK1 | 45.7 | 170.4 | 32.7 | 83.4 | 50.1 | 42.6 |
| WK2 | 49.6 | 156.4 | 32.7 | 83.4 | 49.0 | 54.3 |
| WK3 | 35.8 | 150.0 | 32.7 | 83.4 | 66.9 | 55.4 |
| WK4 | 28.5 | 145.7 | 32.7 | 83.4 | 76.9 | 56.4 |
| WK5 | 48.9 | 167.2 | 32.7 | 83.4 | 47.6 | 46.6 |
| WK6 | 31.0 | 165.8 | 32.7 | 83.4 | 66.3 | 38.9 |
| WK8 | 30.3 | 161.5 | 32.7 | 83.4 | 68.8 | 43.0 |
| WK9 | 26.2 | 161.9 | 32.7 | 83.4 | 72.6 | 40.2 |
| WK10 | 33.2 | 162.9 | 32.7 | 83.4 | 65.3 | 43.1 |
| WK11 | 34.6 | 164.4 | 32.7 | 83.4 | 63.3 | 42.4 |
| WK12 | 31.2 | 157.4 | 32.7 | 83.4 | 69.4 | 47.4 |
| WK13 | 31.9 | 165.1 | 32.7 | 83.4 | 65.7 | 40.1 |
| WK14 | 26.6 | 163.1 | 32.7 | 83.4 | 71.7 | 39.1 |
| WK15 | 46.6 | 168.1 | 32.7 | 83.4 | 49.8 | 44.9 |
| WK16 | 51.4 | 167.7 | 32.7 | 83.4 | 44.8 | 47.1 |
| WK17 | 51.6 | 175.4 | 32.7 | 83.4 | 42.8 | 41.5 |
| The Early Cretaceous (~104–111 Ma) volcanic results from the Qushenla Formation in the Gerze area reported by Chen et al.^41^ | | | | | | |
| GZ23 | 78.3 | 339.6 | 32.7 | 83.4 | 347.0 | 48.2 |
| GZ24-25 | 62.4 | 342.5 | 32.7 | 83.4 | 330.2 | 41.5 |
| GZ26-28 | 77.8 | 329.6 | 32.7 | 83.4 | 347.5 | 45.7 |
| GZ29 | 82.6 | 315.0 | 32.7 | 83.4 | 353.4 | 46.7 |
| GZ30 | 75.4 | 301.3 | 32.7 | 83.4 | 350.5 | 37.3 |
| GZ31 | 76.5 | 305.8 | 32.7 | 83.4 | 350.2 | 39.4 |
| GZ32 | 77.5 | 258.2 | 32.7 | 83.4 | 1.2 | 36.4 |
| GZ33-34 | 78.3 | 294.5 | 32.7 | 83.4 | 353.5 | 39.7 |
| GZ35-36 | 77.6 | 43.8 | 32.7 | 83.4 | 349.4 | 60.8 |
| GZ37 | 76.1 | 52.7 | 32.7 | 83.4 | 350.1 | 62.9 |
| GZ38 | 79.5 | 62.3 | 32.7 | 83.4 | 354.9 | 61.3 |
| GZ39-41 | 82.8 | 51.8 | 32.7 | 83.4 | 355.2 | 58.1 |
| GZ42-43 | 69.7 | 346.7 | 32.7 | 83.4 | 337.0 | 47.0 |
| GZ44-45 | 65.5 | 333.4 | 32.7 | 83.4 | 335.2 | 38.7 |
| The Early Cretaceous (~110–100 Ma) volcanic results in the Lumajiangdongco area reported by Song et al.^43^ | | | | | | |
| lm10 | 9.6 | 152.2 | 32.7 | 83.4 | 86.7 | 40.3 |
| lm14# | 25.8 | 160.3 | 32.7 | 83.4 | 73.7 | 41.7 |
| lm15# | 24.2 | 171.6 | 32.7 | 83.4 | 70.1 | 26.9 |
| lm16# | 22.4 | 148.9 | 32.7 | 83.4 | 82.3 | 51.2 |
| lm17 | 12.8 | 157.5 | 32.7 | 83.4 | 87.4 | 36.3 |
| lm18 | 13.9 | 149.8 | 32.7 | 83.4 | 89.5 | 45.8 |
| lm19 | 24.6 | 153.7 | 32.7 | 83.4 | 77.8 | 47.8 |
| lm20 | 27.9 | 156.0 | 32.7 | 83.4 | 73.4 | 47.2 |
| lm21 | 26.1 | 164.1 | 32.7 | 83.4 | 71.8 | 37.6 |
| lm22 | 14.8 | 155.8 | 32.7 | 83.4 | 86.5 | 39.8 |
| lm23 | 28.4 | 152.8 | 32.7 | 83.4 | 74.2 | 50.4 |
| lm24 | 25.6 | 151.9 | 32.7 | 83.4 | 77.6 | 50.0 |
| lm25 | 19.0 | 148.7 | 32.7 | 83.4 | 86.0 | 49.7 |
| lm26 | 32.6 | 146.3 | 32.7 | 83.4 | 71.8 | 57.3 |
| lm27 | 29.4 | 163.5 | 32.7 | 83.4 | 68.8 | 40.4 |
| lm28 | 33.3 | 159.0 | 32.7 | 83.4 | 66.6 | 46.9 |
| lm29 | 33.9 | 172.8 | 32.7 | 83.4 | 60.8 | 33.0 |
| lm30 | 37.3 | 177.0 | 32.7 | 83.4 | 55.9 | 30.8 |
| lm31 | 18.3 | 139.8 | 32.7 | 83.4 | 88.6 | 57.1 |
| lm32 | 24.5 | 150.5 | 32.7 | 83.4 | 79.3 | 50.8 |
| lm33# | -0.9 | 130.3 | 32.7 | 83.4 | 62.4 | 54.0 |
| lm34# | 21.6 | 146.5 | 32.7 | 83.4 | 84.3 | 53.0 |
| lm36 | 1.7 | 133.0 | 32.7 | 83.4 | 66.9 | 53.6 |
| lm37 | 2.6 | 129.8 | 32.7 | 83.4 | 65.2 | 56.6 |
| lm38 | -0.7 | 129.3 | 32.7 | 83.4 | 61.7 | 54.8 |
| lm39 | 1.7 | 126.8 | 32.7 | 83.4 | 61.9 | 58.2 |
| lm40 | 12.3 | 131.1 | 32.7 | 83.4 | 76.3 | 60.9 |
| lm41 | 8.6 | 134.6 | 32.7 | 83.4 | 74.8 | 56.5 |
| lm42 | 13.3 | 134.6 | 32.7 | 83.4 | 79.8 | 58.9 |
| lm43 | 0.5 | 131.4 | 32.7 | 83.4 | 64.5 | 54.1 |
| lm44 | -3.5 | 134.6 | 32.7 | 83.4 | 63.4 | 48.6 |
| lm45 | 10.5 | 138.4 | 32.7 | 83.4 | 79.4 | 54.4 |
| lm46 | 4.9 | 134.4 | 32.7 | 83.4 | 71.0 | 54.5 |
| lm48 | -6.2 | 135.9 | 32.7 | 83.4 | 62.1 | 45.3 |
| lm49 | 0.8 | 138.1 | 32.7 | 83.4 | 69.8 | 48.6 |
| lm50 | -7.1 | 128.9 | 32.7 | 83.4 | 55.9 | 50.5 |
| lm51 | 3.7 | 135.7 | 32.7 | 83.4 | 70.8 | 52.7 |
| lm53# | 10.7 | 117.9 | 32.7 | 83.4 | 63.2 | 68.3 |
| lm54# | 18.1 | 152.1 | 32.7 | 83.4 | 85.2 | 45.9 |
| lm55# | 14.4 | 129.1 | 32.7 | 83.4 | 77.3 | 63.2 |
| lm56 | 7.5 | 127.3 | 32.7 | 83.4 | 68.1 | 61.1 |
| lm57 | 12.0 | 125.7 | 32.7 | 83.4 | 71.8 | 64.3 |
| **The inclination-only mean of 106 Early Cretaceous palaeomagnetic sites from the western Qiangtang terrane** | | | | | | **43.1 ± 3.4** |
| The western Lhasa terrane | | | | | | |
| The Early Cretaceous (~120–132 Ma) volcanic results from the Qushenla Formation in the Yanhu area reported by Ma et al.^25^ | | | | | | |
| YH1 | 76.9 | 206.8 | 32.7 | 83.4 | 12.0 | 42.9 |
| YH2 | 71.6 | 212.8 | 32.7 | 83.4 | 15.1 | 36.2 |
| YH3 | 56.9 | 187.8 | 32.7 | 83.4 | 34.2 | 35.7 |
| YH4 | 58.5 | 202.1 | 32.7 | 83.4 | 28.2 | 27.3 |
| YH5 | 67.5 | 206.9 | 32.7 | 83.4 | 19.7 | 34.2 |
| YH6 | 67.5 | 209.1 | 32.7 | 83.4 | 19.1 | 33.2 |
| YH7 | 68.1 | 209.2 | 32.7 | 83.4 | 18.6 | 33.8 |
| YH8 | 65.7 | 210.6 | 32.7 | 83.4 | 20.0 | 30.5 |
| YH9 | 62.4 | 204.5 | 32.7 | 83.4 | 24.4 | 30.0 |
| YH10 | 57.3 | 198.5 | 32.7 | 83.4 | 30.5 | 28.5 |
| YH11 | 58.2 | 208.1 | 32.7 | 83.4 | 26.3 | 22.9 |
| YH12 | 59.3 | 206.2 | 32.7 | 83.4 | 26.2 | 25.5 |
| YH13 | 59.2 | 198.0 | 32.7 | 83.4 | 29.1 | 30.7 |
| YH14 | 62.4 | 211.9 | 32.7 | 83.4 | 21.9 | 25.9 |
| YH15 | 61.3 | 204.4 | 32.7 | 83.4 | 25.3 | 28.9 |
| YH16 | 64.5 | 210.1 | 32.7 | 83.4 | 21.0 | 29.4 |
| YH17 | 61.3 | 199.5 | 32.7 | 83.4 | 26.8 | 31.8 |
| YH18 | 64.2 | 215.2 | 32.7 | 83.4 | 19.5 | 26.5 |
| YH19 | 64.7 | 201.7 | 32.7 | 83.4 | 23.4 | 33.8 |
| YH20 | 52.4 | 195.8 | 32.7 | 83.4 | 35.4 | 25.5 |
| YH21 | 62.4 | 208.5 | 32.7 | 83.4 | 23.1 | 27.8 |
| YH22 | 67.5 | 206.9 | 32.7 | 83.4 | 19.7 | 34.2 |
| YH23 | 51.8 | 198.9 | 32.7 | 83.4 | 34.7 | 22.3 |
| YH24 | 61.8 | 193.7 | 32.7 | 83.4 | 28.1 | 35.7 |
| YH25 | 51.0 | 179.1 | 32.7 | 83.4 | 42.3 | 38.3 |
| YH26 | 63.2 | 214.4 | 32.7 | 83.4 | 20.5 | 25.6 |
| YH27 | 65.9 | 201.4 | 32.7 | 83.4 | 22.5 | 35.1 |
| YH28 | 56.3 | 201.0 | 32.7 | 83.4 | 30.4 | 25.6 |
| YH29 | 48.9 | 187.2 | 32.7 | 83.4 | 41.6 | 29.8 |
| YH30 | 68.3 | 195.0 | 32.7 | 83.4 | 21.9 | 40.0 |
| YH31 | 60.8 | 189.0 | 32.7 | 83.4 | 30.3 | 37.8 |
| YH32 | 56.6 | 185.5 | 32.7 | 83.4 | 35.1 | 37.1 |
| YH33 | 59.0 | 179.1 | 32.7 | 83.4 | 34.4 | 42.8 |
| YH34 | 64.5 | 201.5 | 32.7 | 83.4 | 23.6 | 33.8 |
| YH35 | 64.7 | 186.2 | 32.7 | 83.4 | 27.2 | 41.9 |
| YH36 | 59.0 | 171.3 | 32.7 | 83.4 | 35.9 | 47.5 |
| YH37 | 54.7 | 191.6 | 32.7 | 83.4 | 35.0 | 31.1 |
| YH38 | 54.8 | 183.8 | 32.7 | 83.4 | 37.3 | 37.1 |
| YH39 | 66.2 | 165.5 | 32.7 | 83.4 | 28.4 | 52.1 |
| YH42 | 61.8 | 177.8 | 32.7 | 83.4 | 31.8 | 44.9 |
| YH43 | 58.0 | 179.8 | 32.7 | 83.4 | 35.2 | 41.8 |
| YH44 | 58.7 | 176.6 | 32.7 | 83.4 | 35.2 | 44.2 |
| YH45 | 59.1 | 179.9 | 32.7 | 83.4 | 34.1 | 42.3 |
| YH46 | 60.4 | 178.7 | 32.7 | 83.4 | 33.0 | 43.7 |
| YH47 | 60.8 | 186.9 | 32.7 | 83.4 | 30.8 | 39.0 |
| YH48 | 56.0 | 173.6 | 32.7 | 83.4 | 38.7 | 44.9 |
| YH49 | 56.2 | 181.2 | 32.7 | 83.4 | 36.7 | 39.9 |
| YH50 | 63.6 | 180.0 | 32.7 | 83.4 | 29.5 | 44.5 |
| YH51 | 62.6 | 188.3 | 32.7 | 83.4 | 28.7 | 39.4 |
| YH52 | 54.6 | 173.7 | 32.7 | 83.4 | 40.1 | 44.2 |
| YH53 | 52.6 | 179.2 | 32.7 | 83.4 | 40.7 | 39.2 |
| The Early Cretaceous (~110–131 Ma) volcanic results from the Zenong Group in the Cuoqin area reported by Chen et al.^24^ | | | | | | |
| DX1 | 50.4 | 350.1 | 32.7 | 83.4 | 316.4 | 39.9 |
| DX2 | 51.7 | 340.5 | 32.7 | 83.4 | 320.6 | 32.9 |
| DX3 | 46.9 | 354.3 | 32.7 | 83.4 | 311.7 | 41.4 |
| DX4 | 52.5 | 2.0 | 32.7 | 83.4 | 315.8 | 49.5 |
| DX5 | 69.6 | 326.4 | 32.7 | 83.4 | 340.4 | 38.8 |
| DX6 | 65.5 | 333.4 | 32.7 | 83.4 | 335.2 | 38.7 |
| DX7 | 56.7 | 348.8 | 32.7 | 83.4 | 323.0 | 42.3 |
| DX8 | 70.7 | 322.8 | 32.7 | 83.4 | 342.2 | 38.4 |
| DX9 | 56.9 | 345.7 | 32.7 | 83.4 | 324.0 | 40.4 |
| DX10 | 50.9 | 342.0 | 32.7 | 83.4 | 319.4 | 33.5 |
| DX11 | 40.0 | 350.0 | 32.7 | 83.4 | 306.5 | 33.0 |
| DX13 | 62.7 | 352.1 | 32.7 | 83.4 | 328.7 | 46.9 |
| C15 | 56.5 | 332.1 | 32.7 | 83.4 | 327.6 | 30.4 |
| C16 | 61.9 | 331.5 | 32.7 | 83.4 | 332.4 | 34.8 |
| C17 | 60.9 | 328.3 | 32.7 | 83.4 | 332.5 | 32.0 |
| C18 | 69.0 | 300.4 | 32.7 | 83.4 | 347.1 | 28.6 |
| C19 | 56.9 | 348.9 | 32.7 | 83.4 | 323.2 | 42.5 |
| C20 | 56.7 | 342.9 | 32.7 | 83.4 | 324.5 | 38.3 |
| The Early Cretaceous (~117–121 Ma) volcanic results from the Dianzhong Formation in the Cuoqin area reported by Yang et al.^26^ | | | | | | |
| DZ14 | 66.1 | 234.1 | 32.7 | 83.4 | 11.7 | 21.9 |
| DZ15 | 66.1 | 239.3 | 32.7 | 83.4 | 9.7 | 20.4 |
| DZ17 | 79.1 | 204.6 | 32.7 | 83.4 | 10.4 | 45.1 |
| DZ18 | 71.7 | 264.4 | 32.7 | 83.4 | 359.7 | 27.2 |
| DZ19 | 61.9 | 308.7 | 32.7 | 83.4 | 340.0 | 22.0 |
| DZ20 | 62.7 | 303.4 | 32.7 | 83.4 | 342.5 | 20.6 |
| DZ21 | 62.6 | 307.2 | 32.7 | 83.4 | 341.0 | 22.2 |
| DZ22 | 65.4 | 315.8 | 32.7 | 83.4 | 339.9 | 30.0 |
| DZ23 | 65.2 | 307.7 | 32.7 | 83.4 | 342.4 | 26.1 |
| DZ24 | 70.3 | 315.9 | 32.7 | 83.4 | 343.5 | 35.5 |
| DZ25 | 70.8 | 307.9 | 32.7 | 83.4 | 346.0 | 33.4 |
| DZ26 | 63.7 | 319.3 | 32.7 | 83.4 | 337.6 | 29.8 |
| The Early Cretaceous (~120–106 Ma) volcanic and red beds results from the Risong Formation in the Gerze area reported by Wang et al.^29^ | | | | | | |
| WM1 | 34.9 | 149.5 | 32.7 | 83.4 | 68.1 | 55.5 |
| WM2 | 34.4 | 160.8 | 32.7 | 83.4 | 64.8 | 45.8 |
| WM3 | 37.3 | 159.3 | 32.7 | 83.4 | 62.3 | 48.4 |
| WM4 | 35.3 | 153.8 | 32.7 | 83.4 | 66.2 | 52.3 |
| WM5 | 38.7 | 158.9 | 32.7 | 83.4 | 60.9 | 49.3 |
| WM6 | 36.6 | 159.0 | 32.7 | 83.4 | 63.1 | 48.4 |
| WM7 | 43.4 | 159.7 | 32.7 | 83.4 | 55.5 | 50.3 |
| WM8 | 32.8 | 158.6 | 32.7 | 83.4 | 67.3 | 47.1 |
| WM9 | 34.3 | 162.0 | 32.7 | 83.4 | 64.5 | 44.6 |
| WM10 | 42.7 | 161.3 | 32.7 | 83.4 | 55.8 | 48.8 |
| WM11 | 38.5 | 161.5 | 32.7 | 83.4 | 60.3 | 47.0 |
| WM12 | 40.1 | 168.5 | 32.7 | 83.4 | 56.4 | 41.4 |
| WM13 | 37.4 | 167.0 | 32.7 | 83.4 | 59.6 | 41.3 |
| WM14 | 35.5 | 169.7 | 32.7 | 83.4 | 60.5 | 37.5 |
| WM15 | 44.3 | 166.6 | 32.7 | 83.4 | 52.7 | 45.1 |
| WM16 | 42.1 | 163.7 | 32.7 | 83.4 | 55.8 | 46.6 |
| WM17 | 40.7 | 163.1 | 32.7 | 83.4 | 57.5 | 46.5 |
| WM35 | 43.7 | 152.7 | 32.7 | 83.4 | 56.8 | 55.5 |
| WM36 | 35.3 | 153.8 | 32.7 | 83.4 | 66.2 | 52.3 |
| WM37 | 42.9 | 156.5 | 32.7 | 83.4 | 56.8 | 52.6 |
| WM38 | 46.7 | 155.8 | 32.7 | 83.4 | 52.6 | 54.1 |
| WM39 | 37.6 | 159.6 | 32.7 | 83.4 | 61.9 | 48.2 |
| WM40 | 38.7 | 153.2 | 32.7 | 83.4 | 62.5 | 53.8 |
| WM41 | 34.3 | 152.3 | 32.7 | 83.4 | 67.9 | 53.1 |
| WM18 | 69.8 | 234.9 | 32.7 | 83.4 | 9.8 | 27.5 |
| WM19 | 71.7 | 280.2 | 32.7 | 83.4 | 354.6 | 28.3 |
| WM20 | 77.2 | 228.8 | 32.7 | 83.4 | 7.8 | 38.8 |
| WM21 | 69.6 | 273.5 | 32.7 | 83.4 | 356.4 | 24.0 |
| WM46 | 79.7 | 214.6 | 32.7 | 83.4 | 8.6 | 43.8 |
| WM47 | 78.2 | 274.5 | 32.7 | 83.4 | 357.6 | 37.7 |
| WM22 | 76.0 | 283.8 | 32.7 | 83.4 | 354.9 | 35.3 |
| WM23 | 70.7 | 300.9 | 32.7 | 83.4 | 347.9 | 31.1 |
| WM24 | 75.5 | 288.6 | 32.7 | 83.4 | 353.5 | 35.2 |
| WM25 | 78.9 | 272.6 | 32.7 | 83.4 | 358.1 | 38.6 |
| WM26 | 77.3 | 285.6 | 32.7 | 83.4 | 354.9 | 37.3 |
| WM48 | 78.6 | 273.4 | 32.7 | 83.4 | 357.9 | 38.2 |
| WM49 | 75.6 | 271.8 | 32.7 | 83.4 | 357.8 | 33.7 |
| WM50 | 76.7 | 293.2 | 32.7 | 83.4 | 353.0 | 37.5 |
| WM51 | 74.9 | 246.4 | 32.7 | 83.4 | 4.6 | 33.3 |
| WM52 | 75.7 | 269.6 | 32.7 | 83.4 | 358.4 | 33.7 |
| WM27 | 73.1 | 318.4 | 32.7 | 83.4 | 345.1 | 39.1 |
| WM28 | 71.3 | 275.9 | 32.7 | 83.4 | 355.9 | 27.1 |
| WM29 | 70.9 | 279.9 | 32.7 | 83.4 | 354.5 | 27.0 |
| WM30 | 78.0 | 310.5 | 32.7 | 83.4 | 350.4 | 41.9 |
| WM31 | 75.2 | 266.4 | 32.7 | 83.4 | 359.2 | 32.9 |
| WM32 | 67.7 | 278.8 | 32.7 | 83.4 | 354.1 | 21.4 |
| WM33 | 67.3 | 290.6 | 32.7 | 83.4 | 349.6 | 23.2 |
| WM34 | 74.4 | 272.3 | 32.7 | 83.4 | 357.5 | 31.9 |
| WM42 | 70.5 | 301.4 | 32.7 | 83.4 | 347.6 | 31.0 |
| WM43 | 75.0 | 273.7 | 32.7 | 83.4 | 357.2 | 32.9 |
| WM44 | 74.2 | 286.7 | 32.7 | 83.4 | 353.5 | 33.1 |
| WM45 | 66.9 | 299.4 | 32.7 | 83.4 | 346.3 | 25.3 |
| WM53 | 77.5 | 275.6 | 32.7 | 83.4 | 357.2 | 36.7 |
| WM54 | 79.9 | 268.7 | 32.7 | 83.4 | 359.0 | 39.8 |
| WM55 | 77.8 | 270.1 | 32.7 | 83.4 | 358.5 | 36.9 |
| WM56 | 76.5 | 270.7 | 32.7 | 83.4 | 358.2 | 35.0 |
| The Early Cretaceous (~116–113 Ma) Zenong Group volcanic rocks and Jiega Formation limestone results from the Shiquanhe area reported by Bian et al.^27^ | | | | | | |
| ZN1 | 71.5 | 329.2 | 32.7 | 83.4 | 341.6 | 41.4 |
| ZN2 | 76.0 | 317.6 | 32.7 | 83.4 | 347.6 | 41.5 |
| ZN3 | 63.7 | 323.8 | 32.7 | 83.4 | 336.2 | 32.2 |
| ZN4 | 75.5 | 268.4 | 32.7 | 83.4 | 358.7 | 33.4 |
| ZN5 | 71.0 | 325.4 | 32.7 | 83.4 | 341.9 | 39.6 |
| ZN6 | 65.0 | 335.8 | 32.7 | 83.4 | 334.2 | 39.6 |
| ZN7 | 65.4 | 321.5 | 32.7 | 83.4 | 338.2 | 32.7 |
| ZN8 | 48.9 | 335.4 | 32.7 | 83.4 | 320.0 | 25.9 |
| ZN9 | 57.0 | 332.5 | 32.7 | 83.4 | 327.9 | 31.2 |
| ZN10 | 54.7 | 331.6 | 32.7 | 83.4 | 326.2 | 28.3 |
| ZN11 | 62.7 | 325.4 | 32.7 | 83.4 | 334.9 | 32.1 |
| ZN12 | 65.6 | 329.4 | 32.7 | 83.4 | 336.2 | 36.8 |
| ZN13 | 63.2 | 334.9 | 32.7 | 83.4 | 332.7 | 37.8 |
| ZN14 | 62.4 | 334.0 | 32.7 | 83.4 | 332.2 | 36.7 |
| ZN15 | 62.1 | 332.8 | 32.7 | 83.4 | 332.3 | 35.8 |
| ZN16 | 68.7 | 328.7 | 32.7 | 83.4 | 339.1 | 39.0 |
| ZN17 | 61.3 | 332.7 | 32.7 | 83.4 | 331.6 | 35.1 |
| ZN18 | 62.9 | 330.2 | 32.7 | 83.4 | 333.7 | 35.0 |
| ZN19 | 64.6 | 327.1 | 32.7 | 83.4 | 336.0 | 34.8 |
| ZN20 | 63.4 | 324.3 | 32.7 | 83.4 | 335.8 | 32.2 |
| ZN21 | 60.0 | 329.4 | 32.7 | 83.4 | 331.4 | 31.9 |
| ZN22 | 69.1 | 311.3 | 32.7 | 83.4 | 343.9 | 32.5 |
| ZN23 | 70.9 | 297.2 | 32.7 | 83.4 | 349.1 | 30.4 |
| ZZ8 | 78.4 | 295.5 | 32.7 | 83.4 | 353.3 | 39.9 |
| ZZ9 | 67.1 | 276.1 | 32.7 | 83.4 | 355.0 | 19.9 |
| ZZ10 | 54.7 | 333.6 | 32.7 | 83.4 | 325.5 | 29.9 |
| ZZ11 | 78.2 | 259.0 | 32.7 | 83.4 | 1.0 | 37.4 |
| ZZ12 | 76.2 | 260.4 | 32.7 | 83.4 | 0.8 | 34.4 |
| ZZ13 | 82.3 | 232.9 | 32.7 | 83.4 | 4.3 | 44.3 |
| **The inclination-only mean of 166 Early Cretaceous palaeomagnetic sites from the western Qiangtang terrane** | | | | | | **36.9 ± 1.6** |

*Notes:* Plat and Plon, latitude and longitude of pole; Rlat and Rlon, latitude and longitude of reference point; Dr and Ir, declination and inclination calculated for the reference point (32.7°N, 83.4°E). The inclination-only mean is calculated following the method of Arason and Levi.^73^
